# Insulin-like peptides activate egg formation in the Asian malaria mosquito *Anopheles stephensi*

**DOI:** 10.1186/s13071-025-07036-y

**Published:** 2025-10-07

**Authors:** Benjamin L. Phipps, Mark R. Brown, Michael R. Strand

**Affiliations:** 1 Department of Genetics, University of Georgia, Athens, GA 30602 USA; 2 Department of Entomology, University of Georgia, Athens, GA 30602 USA; 3 Center for Tropical and Emerging Global Diseases, University of Georgia, Athens, GA 30602 USA

**Keywords:** Anophilinae, Hormone, Gonotrophic cycle, Mating, Vitellogenesis

## Abstract

**Background:**

The mosquito family Culicidae diverged into the subfamilies Anophelinae and Culicinae approximately 179 million years ago. Most female mosquitoes are anautogenous and must blood-feed on a vertebrate to produce eggs. Regulation of egg-producing gonotrophic cycles is best understood in the culicine *Aedes aegypti.* Anopheline mosquitoes encode all of the hormones that regulate gonotrophic cycles in *Ae. aegypti,* but the processes regulating egg formation may not be fully similar. In this study, we conducted experiments that compared egg formation in *Anopheles stephensi* to prior findings reported for *Ae. aegypti.*

**Methods:**

Assays for yolk deposition into oocytes, ovary ecdysteroidogenesis, *vitellogenin* expression, nutrient storage and oviposition were used to characterize gonotrophic cycles in *An. stephensi* females that were mated or unmated.

**Results:**

Yolk deposition into oocytes depended on the release of hormones produced in the head*.* Two insulin-like peptides, *An. stephensi* insulin-like peptide hormone 3 (AsILP3) and AsILP4, stimulated the vitellogenic phase in *An. stephensi,* as measured by several different assays, whereas ovary ecdysteroidogenic hormone (OEH) showed no stimulatory activity*.* Nutrient stores were lower in *An. stephensi* than *Ae. aegypti*, which was associated with females also being unresponsive to AsILP3 stimulation in the absence of a blood meal. *Anopheles stephensi* males transferred ecdysteroids (ECDs) to females, which was associated with mated females producing and laying more eggs than unmated females. However, mated and unmated females did not show differences in ECD production by the ovaries or *vitellogenin* expression at the messenger RNA level by the fat body. Most females that mated before consuming a first blood meal oviposited while most unmated females did not. Mating after consuming a first blood meal did not rescue oviposition. However, females that reabsorbed eggs and consumed a second blood meal did oviposit.

**Conclusions:**

Regulation of gonotrophic cycles in *An. stephensi* shares some features with *Ae. aegypti* but also exhibits differences.

**Graphical abstract:**

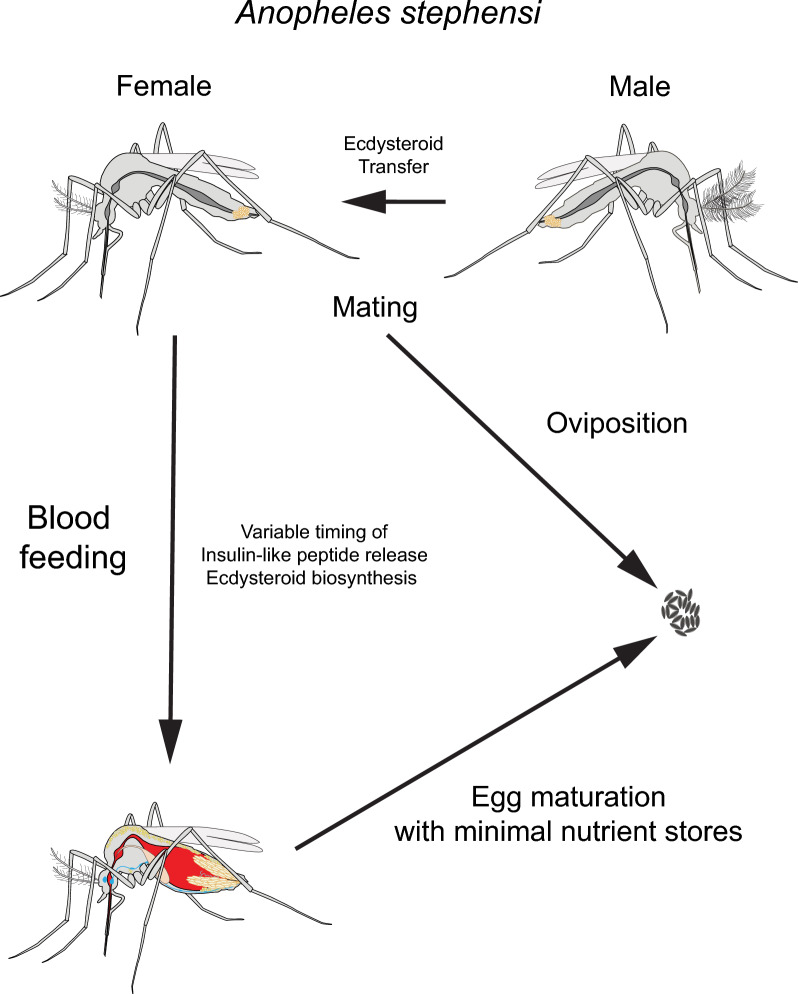

**Supplementary Information:**

The online version contains supplementary material available at 10.1186/s13071-025-07036-y.

## Background

An estimated 3600 mosquito species (family Culicidae) exist worldwide, of which approximately 300 are disease vectors [1]. Adults of both sexes consume water and sugar that provide energy for maintenance functions and flight [2, 3]. However, most species are also anautogenous, which means females must blood-feed on a human or other vertebrate host to produce eggs [4]. Most studied species produce one clutch of eggs per blood meal consumed [5]. Sequential blood meals and gonotrophic (egg-producing) cycles over a lifespan of several weeks underlie how vector species acquire and transmit pathogens between hosts. Thus, the mechanisms regulating egg formation are also important to vector biology.

Phylogenomic data strongly support the monophyly of the Culicidae, which diverged into two subfamilies, the Anophelinae and Culicinae, approximately 179 million years ago [6]. Regulation of egg formation has been most studied and is best understood in *Aedes aegypti*, which is a human disease vector in the Culicinae (tribe Aedini) [7]. *Aedes aegypti* females emerge from the pupal stage in a previtellogenic phase in which ovaries contain 100–150 primary follicles. Each primary follicle consists of an oocyte and nurse cells that are surrounded by somatic follicle cells [8]. Primary follicles increase in size 1–2 days post-emergence (PE) under control of juvenile hormone (JH) [9]. Previtellogenic females consume sugar and water and begin to seek hosts for blood-feeding 3–4 days PE [2, 10, 11]. However, primary follicles remain developmentally arrested until a female consumes a blood meal, which primarily consists of protein by dry weight [5].

**Fig. 1:**
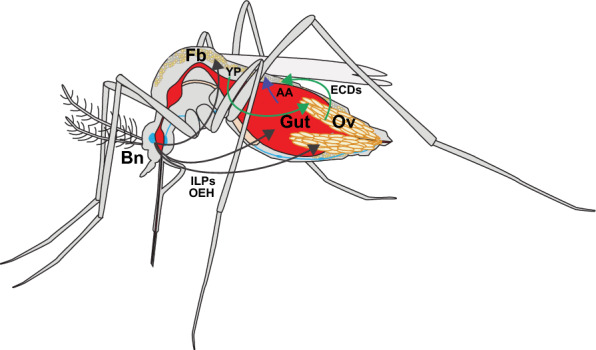
Schematic showing key processes regulating the vitellogenic phase after *Aedes aegypti* females consume a blood meal. See text for a full description of the events that activate the vitellogenic phase which culminates in maturation of eggs that females lay by 72 h post-blood meal. AA, Amino acid; Bn, brain; ECDs, ecdysteroids; Fb, fat body; ILPs, insulin-like peptides; OEH, ovary ecdysteroidogenic hormone; Ov, ovaries; Yp, yolk protein

The vitellogenic phase in *Ae. aegypti* begins by 2 h post-blood meal (PBM) with release of insulin-like peptides (ILPs) and ovary ecdysteroidogenic hormone (OEH) from brain medial neurosecretory cells (mNSCs) [12, 13] (Fig. 1). ILPs and OEH are structurally distinct but bind closely related tyrosine receptor kinases named the insulin receptor (IR) and OEH receptor (OEHR) [14–16]. Activation of the IR and OEHR both induce insulin-insulin growth factor-like signaling (IIS) [13, 17]. IIS in the ovaries activates primary follicles and synthesis of ecdysteroids (ECDs) that are converted to 20-hydroxyecdysone (20E) [8, 16–18] (Fig. 1). Stored nutrients in the fat body are mobilized under regulation of adipokinetic hormone and JH [19–21], while 20E, IIS and nutrient signaling via the target of rapamycin (TOR) pathway regulate yolk production by the fat body, blood meal digestion and replenishment of nutrient stores [13, 19, 22–26] (Fig. 1). Yolk uptake by oocytes followed by chorion (eggshell) formation produces mature eggs 48–60 h PBM that females lay by 72 h PBM [8, 22]. Secondary follicles in the ovaries transition to primary follicles in association with egg laying, which is followed by entry into another previtellogenic phase that persists until a female consumes another blood meal [27].

A number of anopheline species are important vectors of human malaria [28]. *Anopheles stephensi* and *Anopheles gambiae* encode several ILP family members and OEH while IIS has functions in metabolic regulation [29–34]. The ovaries of *An. stephensi* produce ECDs in response to ILPs, and 20E titers increase 16–30 h PBM under standard conditions before females lay mature eggs 48–72 h PBM [33, 35]. In contrast, the ancient divergence time of culicines and anophelines together with differences in certain life history traits suggest that the regulation of gonotrophic cycles may differ in four respects. First, *Ae. aegypti* females deposit no yolk into oocytes if decapitated 1 h PBM; rather they deposit near normal amounts of yolk by 48 h PBM if decapitated at 2 h PBM. This finding provides the experimental evidence that *Ae. aegypti* females release sufficient quantities of ILPs and OEH from brain mNSCs within 2 h of blood-feeding to activate the vitellogenic phase [13]. In contrast, no studies have been conducted in anopheline mosquitoes to determine if or when sufficient quantities of hormones produced in the head (hereafter head-produced) are released to stimulate egg formation after blood-feeding. Second, *Ae. aegypti* encodes eight ILPs, of which five (AaILP1, 3, 4, 7, 8) plus OEH are specifically expressed in brain mNSCs [12, 13]. The injection of a single 20 pmol dose of each mNSC-specific ILP or OEH into females decapitated within 1 h PBM rescue the vitellogenic phase, as evidenced by ovaries producing ECD, the fat body expressing yolk-producing gene products and oocytes packaging normal amounts of yolk by 48 h PBM [8, 13]. *Anopheles stephensi* and *An. gambiae* encode five ILPs plus OEH, with prior results showing that *An. stephensi* ILP3 (AsILP3), AsILP4 and *An. gambiae* OEH (AgOEH) are produced in mNSCs [29, 32, 33]. In contrast, whether head-produced ILPs or OEH fully activate the vitellogenic phase in anopheline mosquitoes is unknown.

Third, several studies report differences in nutrient acquisition during the larval stage that result in culicines emerging as adults with larger nutrient stores than anophelines [36–38]. Culicines can also increase nutrient stores by sugar-feeding or replenish stores by blood-feeding [19, 20]. Larger nutrient reserves likely facilitate nutrient provisioning to eggs that in a number of culicine species survive adverse conditions through the evolution of an obligate diapause or quiescence [39–41]. Larger nutrient reserves also enable *Ae. aegypti* and other culicines to mature eggs in response to ILPs and OEH without blood-feeding, which likely underlies blood meal-independent egg formation (autogeny) evolving several times in the Culicinae [42, 43]. Few studies have assessed how sugar- or blood-feeding affects nutrient stores in anopheline mosquitoes. However, anopheline mosquitoes are generally unknown to lay diapausing or quiescing eggs that usually hatch rapidly after being laid on water [44]. Autogeny is also unknown in the Anophelinae but whether this is due to head-produced hormones not being able to stimulate egg formation in the absence of a blood meal is unstudied. Fourth, male *Anopheles* spp. transfer ECDs to females when mating via accessary secretions that form a mating plug, whereas *Ae. aegypti* and other culicines do not [45–48]. Transferred ECDs increase egg production after *An. gambiae* females blood-feed [49], but underlying mechanisms for this response remain unclear.

In the study reported here, we conducted experiments that addressed the four study areas summarized above in *An. stephensi*. Our results identify differences in the timing of the release and activity of ILPs and OEH in mated *An. stephensi* when compared to *Ae. aegypti*. Male *An. stephensi* transferred ECD to females during mating, which was associated with increased yolk deposition per oocyte when compared to unmated females and oviposition. Mating before blood-feeding results in most *An. stephensi* females ovipositing. In contrast, neither mating nor injection of ECD after blood-feeding rescued oviposition.

## Methods

### Mosquitoes

The *An. stephensi* Indian strain used in the study originated from the colony maintained at the Walter Reed Army Institute of Research, (Silver Spring, MD, USA) and was reared in an insectary at 28 °C and 70% relative humidity on a 12:12 h light:dark photoperiod [27]. After hatching, larvae (200/l in deionized water) were fed ground TetraMin® fish food (Tetra, Melle, Germany) daily until pupation. Pupae were collected and placed in cages where adults emerged and mated. Adults were provided ad libitum access to water and a sugar solution containing 4% sucrose and 4% fructose in water. Eggs for colony maintenance were obtained by feeding 3- to 6-day-old adult females defibrinated rabbit blood (Hemostat Laboratories, Dixon, CA, USA) in membrane feeders. To obtain virgin adults, individual pupae were placed in wells of 96-well plates containing water, and adults were then segregated by sex after eclosion. Females used in all experiments were of similar size (approx. 2.5–3.0 mm) as measured by forewing length [50], which is widely used as a size measure for adult mosquitoes. Unless otherwise stated, all females used in experiments were mated in cages where pupae of both sexes were allowed to emerge. For most experiments, 4 day PE females were fed rabbit blood using artificial membrane feeders, similar to the general colony. However, unavailability of rabbit blood from our commercial source at the time the mating assays were conducted led us to use voluntary blood-feeding on the arm of one of the authors (BLP). For one set of experiments, we used the University of Georgia (UGAL) strain of *Ae. aegypti,* which was reared in our laboratory at the same temperature and photoperiod as *An. stephensi. Aedes aegypti* larvae were fed and adults were maintained as previously detailed [5].

### Decapitation and hormone injection assays

The timing of neurohormone release and activation of the vitellogenic phase was assessed by decapitating adult females 1–24 h PBM; intact (not decapitated) females served as a positive control. Ovaries were then explanted from females at 48 h PBM, and the amount of yolk present in oocytes was measured using the same methods that have been carefully described for *Ae. aegypti* [12, 50]. In brief, yolk deposition per oocyte in a female is near fully uniform across all primary follicles at 48 h PBM. Thus, yolk in three primary follicles per ovary was measured lengthwise using an ocular micrometer on a dissecting microscope; the average length was then used as the amount of yolk per oocyte [50].

We previously reported the synthesis of *Ae. aegypti* ILP3 (AaILP3) and AaILP4 as separate B and A chains followed by cross-linking and purification [12]. *Anopheles stephensi* ILP3 (AsILP3) and AsILP4 used in this study were similarly synthesized with > 80% purity by CPC Scientific (Sunnydale, CA, USA) [33]. We also produced and purified *An. stephensi* OEH (AsOEH; approx. 18 kDa) as a recombinant protein in *Escherichia coli* from complementary DNA (cDNA; VectorBase Accession ASTE004312) using the same protocol previously used to generate AaOEH [16]. AsILP3, AsILP4, AsOEH and commercially purchased 20E (Sigma, Saint Louis, MO, USA) were frozen as aliquots of 200 pmol/μl in water and 2 µg/µl, respectively.

Four day PE *An. stephensi* females were decapitated 1 h or 12 h PBM and injected with either 20 pmol of AsILP3, AsILP4, AsOEH or AaOEH, 1 mmol of 20E or 20 pmol of AsILP3 + 1 mmol of 20E, in a 0.25 μl volume of physiological *Aedes* saline (9 g NaCl, 0.2 g CaCl_2_, 0.2 KCl, 0.1 NaHCO_3_ per liter of ultrapure water). These doses were used because they are consistent with previous measures of hemolymph ILP and OEH titers after blood-feeding, while these doses of ILP, OEH and 20E strongly activate the vitellogenic phase in *Ae. aegypti* [12, 13, 25, 26, 33]. Doses of 20 pmol of AsILP3 and AsILP4 were previously shown to activate ECD production by *An. stephensi* ovaries [33]. For select experiments, we also injected decapitated *An. stephensi* females with 40 pmol of AsOEH or 2 mmol of 20E. Decapitated females injected with 0.25 μl of saline alone served as a negative control, while intact, blood-fed females that were not injected with anything served as a positive control. Mated, 4-day PE *Ae. aegypti* females were blood-fed to repletion, decapitated at 1 h or 12 h PBM and immediately thereafter injected with 20 pmol of AsOEH in 0.25 μl of saline, 20 pmol of AaOEH in 0.25 μl of saline or 0.25 μl saline alone. This was followed by measuring the amount of yolk per oocyte at 48 h PBM. Intact, blood-fed females that were not injected with anything served as a positive control. Each hormone in saline or saline only was injected into females that were first chilled on ice for 5 min using borosilicate glass capillaries (outside diameter 1 mm, inside diameter 0.78 mm) (World Precision Instruments, Sarasota, FL, USA) that were pulled into needles on a Flaming/Brown micropipette puller (Sutter Instruments, Novato, CA, USA). Needles were manually calibrated into 0.25 μl volumes, mounted to a micromanipulator (Narashige International, Amityville, NY, USA) and connected by tubing to a 10-ml glass syringe (Corning Glass, Corning, NY, USA). Placement of the needle and micromanipulator under a stereomicroscope (Leica, Wetzlar, Germany) then enabled manual injection of the correct dose of each treatment by piercing the thorax of each female with the needle tip. Decapitated females were held in humidified plastic cups; intact females were held in the same type of cup but were also provided water-soaked cotton wicks.

### Transcript abundance of the IR and vitellogenin1

Gene-specific primers were synthesized by IDT (Integrated DNA Technologies, Coralville, IA, USA) for the* An. stephensi* insulin receptor mRNA (*Asir*; VectorBase Accession ASTEI02289: forward primer 5’-CGTAACGGAGTCGGAGAGTG-3’, reverse primer 5’-CGCCGTTTTCGACTGGATTT-3) and *An. stephensi* vitellogenin1 (*Asvg*; VectorBase Accession ASTE003745: forward primer 5’-TGGTACAACTACACCATCCAGTC-3’, reverse primer 5’-CTTGATTTCGTTGAGGGTCATGT-3’). Whole abdomens from previtellogenic phase females 0–4 PE were collected by dissection and individually placed in Trizol reagent (Thermo Fisher Scientific, Waltham, MA, USA). Ovaries (6 pairs per tube) and pelts (abdomen body walls containing the fat body with the digestive tract and ovaries removed, 3 per tube) were dissected from *An. stephensi* females 0–72 h PBM and placed in Trizol. Four-day-old PE females were also blood-fed, decapitated within 1 h and immediately injected with 20E or AsILP3 alone or together, with untreated, intact females serving as a positive control. Pelts were collected from these females at 0, 6 and 12 h PBM and placed in Trizol reagent (3 per tube). Total RNA was extracted from all samples per the manufacturer’s instructions, followed by reverse transcription of a 1-µg sample using the iScript cDNA synthesis kit (Bio-Rad Laboratories, Hercules, CA, USA). *Asir* or *Asvg* transcript abundance was quantified by quantitative reverse transcriptase-PCR (qRT-PCR) assays that measured the total copy number of each transcript of interest in a given sample using previously reported methods [13]. In brief, we first generated the cDNA template from ovaries or pelts from non-blood-fed 4 day PE females. Total RNA was synthesized into cDNA, which was then used as template in the qRT-PCR assays that used the above primers corresponding to 100- to 200-bp regions of each targeted gene. Each PCR product was first cloned into PCR®2.1 TOPO® TA (Invitrogen, Carlsbad, CA, USA), which was then transformed into NEB-10β-competent *E. coli* cells (New England Biolabs, Inc. [NEB], Ipswich, MA, USA). The resulting plasmid DNA was extracted using a GeneJET Plasmid Miniprep Kit (Thermo Fisher Scientific), and the *Asir* or *Asvg* insert was confirmed by sequencing (Macrogen, Seoul, Republic of Korea). Absolute standard curves were then generated by serially diluting each plasmid (10^2^ to 10^7^ copies) using iQ SYBR Green Supermix (Bio-Rad Laboratories) and specific qPCR primers. For each gene, time point and treatment, samples from at least three female mosquitoes were examined (i.e. 3 biological replicates) while each qRT-PCR assay was run in quadruplicate (4 technical replicates) on a Rotor-Gene Q real-time PCR cycler (Qiagen-USA, Germantown MD, USA) under the following conditions: initial denaturation at 95 °C for 10 min, followed by 30 cycles with denaturation at 95 °C for 10 s, annealing at 60 °C for 45 s and extension at 72 °C for 20 s.

### ECD production by ovaries in the presence and absence of inhibitors

The production of ECD by ovaries was measured using a previously developed ex vivo assay and enzyme-linked immunoassay (EIA) [51]. In brief, ovaries were explanted from 4 day PE females and placed in Sf-900 medium (HyClone, Logan, UT, USA) at 28 °C for 6 h with or without 20 pmol AsILP3 (2 ovary pairs in 60 µl in triplicate wells, for 3 sets). Other assays included 17 µM OSI-906 (LC Laboratories, Woburn, MA, USA), which is a potent inhibitor of the IR in *Ae. aegypti,* 50 pM of Torin2 (Cayman, Ann Arbor, MI, USA), which is a second-generation inhibitor of TORC1 and TORC2 in *Ae. aegypti* and other organisms [8] or 170 µM rapamycin (Sigma), which is a first-generation inhibitor of the TOR pathway in mosquitoes and other organisms [52]. Stock solutions of each of these inhibitors were made in dimethylsulfoxide (DMSO). Medium was collected for ovary cultures and stored at − 20 °C followed by EIA analysis following the detailed protocol of McKinney et al. [51].

### Triacylglycerol and glycogen stores

Pelts were collected by dissection in phosphate-buffered saline (PBS; 1.44 g Na_2_HPO_4_, 0.2 KCl, 0.24 g KH_2_PO_4_ in ultrapure water pH 7.2) from 0 to 4 day old PE females. Four day PE females were also blood-fed, followed by the collection of pelts at 24-h intervals up to 96 h PBM. Each biological sample consisted of two pelts that were homogenized in 100 µl of PBS containing 0.5% Tween-20 and incubated at 70 °C for 5 min. Pelt samples were centrifuged at 3000 *g* for 1 min and 15,900 *g* for 3 min followed by the transfer of 10 µl from each sample to individual wells of 96-well plates (Corning, Cambridge, MA, USA). After adding 100 µl of TAG reagent (Thermo Fisher Scientific) and gentle mixing, samples were incubated for 10 min at room temperature, and absorbance was measured with a Synergy plate reader (Agilent BioTek, Winooski, VT, USA) at 530 nm. A range of TAG standards (MedTest Dx, Canton, MI, USA) was included in the wells of each plate and used to calculate experimental values from a regression line. Glycogen levels were measured using a Glycogen Assay Kit (Cayman, Ann Arbor, MI, USA) per instructions from the manufacturer. Briefly, two pelts were homogenized in 100 µl of diluent assay buffer and then centrifuged at 60 *g* for 10 min. Sample supernatants (10-µl aliquots) were transferred to wells in 96-well plates followed first by the addition of hydrolysis enzyme solution (50 µl) for a 30-min incubation at 37 °C and then by the addition of developer solution (150 µl) for 15 min at 37 °C. Plate wells were read with the Synergy plate reader as described above with an excitation wavelength of 530 nm and an emission wavelength of 585 nm. Glycogen standards in wells of the same plate were similarly set up and read to calculate experimental values as described above.

### Assays comparing unmated and mated females

Ecdysteroids were measured in reproductive tract and carcass extracts of 4 day PE unmated males, 4 day PE unmated females and 4 day PE mated females at 1, 6, 12 or 24 h after mating with a male who had not previously mated. Females were mated by placing approximately 15 females in a 31 × 31 × 31-cm cage with approximately  50 virgin males at dusk for 30 min; dissection and inspection of the spermatheca in preliminary assays confirmed that all females were mated after this period. Reproductive tracts (RTs) from unmated males and females were collected by removing the last three abdominal segments which contained the RT (testis + accessory glands [males] or ovaries, lateral oviducts, common oviduct and spermatheca [females]). RTs were immediately placed after collection in 100 µl of PBS (1 per tube), briefly sonicated and centrifuged, followed by transfer of the supernatant, referred to as the RT extract, to a new tube. The rest of the body was crushed in a small chamber with 100 µl of 1× PBS followed by transfer of the supernatant, referred to as the carcass extract, to a new tube. All samples (6–9 replicates for each time) were frozen at − 20 °C and later thawed to quantify ecdysteroids by the EIA as described above. We next blood-fed mated 4 day PE females from the general culture or 4 day PE unmated females. Only females that blood-fed to repletion were used in these assays. One cohort of these females was used to measure yolk deposition per oocyte at 48 h PBM as described above. Other cohorts were used to measure ECD production by ovaries and *Asvg* transcript abundance in the fat body of unmated and mated females at 1, 12, 18, 24, 36, and 48 after blood-feeding. Two pairs of ovaries were dissected at the above times from females and placed into Sf-900 medium for 6 h followed by processing for EIA as described above. Transcript abundance of *Asvg* was measured by isolating pelts followed by isolation of total RNA and RT-qPCR assays as described above. Lastly, experiments were conducted that examined oviposition. First, 4 day PE mated and unmated females were blood-fed to repletion, followed at 48 h PBM by placing females individually in chambers with wet filter paper as an oviposition substrate; the number of eggs each individual laid was counted at 96 h PBM. This was followed by allowing 4 day PE unmated females to blood-feed to repletion, followed by transfer of approximately 15 females to 31 × 31 × 31-cm cages containing approximately 50 virgin males, water and a sugar solution. These females were held with males for 72 h, followed by the transfer of females to chambers with wet filter paper where they were held for an additional 72 h. The number of eggs laid by each female was then counted, followed by the dissection of each female in PBS. The spermatheca of each female was then transferred to a slide, inspected by phase contrast microscopy and scored as mated if sperm was present. Four day PE mated females that were blood-fed to repletion, and then transferred to oviposition chambers at 72 h PBM for oviposition and inspection of the spermatheca served as the control treatment. Unmated 4 day PE females were also allowed to blood-feed to repletion followed by the injection of 1 mmol of 20E in 0.25 μl of saline or the injection of the same volume of saline alone at 48 h PBM. Females were then transferred individually to chambers with wet filter paper as an oviposition substrate; the number of eggs each individual laid was counted at 96 h PBM. Eggs laid by these females were compared to those of mated, 4 day PE females that similarly were allowed to blood-feed to repletion and oviposit in individual chambers.

### Data analysis

All statistical analyses were conducted using GraphPad Prism v10.2.2 (GraphPad Software, La Jolla, CA, USA). For each experiment, treatment groups were analyzed for normality using Shapiro–Wilk tests and for significant differences in standard deviation between groups using Brown-Forsythe and Bartlett’s tests. If data were normally distributed and standard deviations were similar between groups, unpaired t-tests were performed for experiments with two treatment groups. One-way analyses of variance (ANOVAs) were performed when there were more than two groups and a single independent variable, followed by a post-hoc Tukey’s multiple comparison test that compared all treatments to one another or a Dunn’s test that compared treatments to a designated control. If data were not normally distributed and/or standard deviations were significantly different between treatment groups, Mann–Whitney tests were performed for experiments with two treatment groups, and Kruskal–Wallis tests were performed when there were more than two groups and a single independent variable, followed by post-hoc Dunn’s tests. The relationship between female body size and yolk deposition per oocyte was assessed using Spearman’s rank correlation coefficient.

## Results

### Head-dependent activation of egg development is slower in *An. stephensi* than in *Ae. aegypti*

**Fig. 2:**
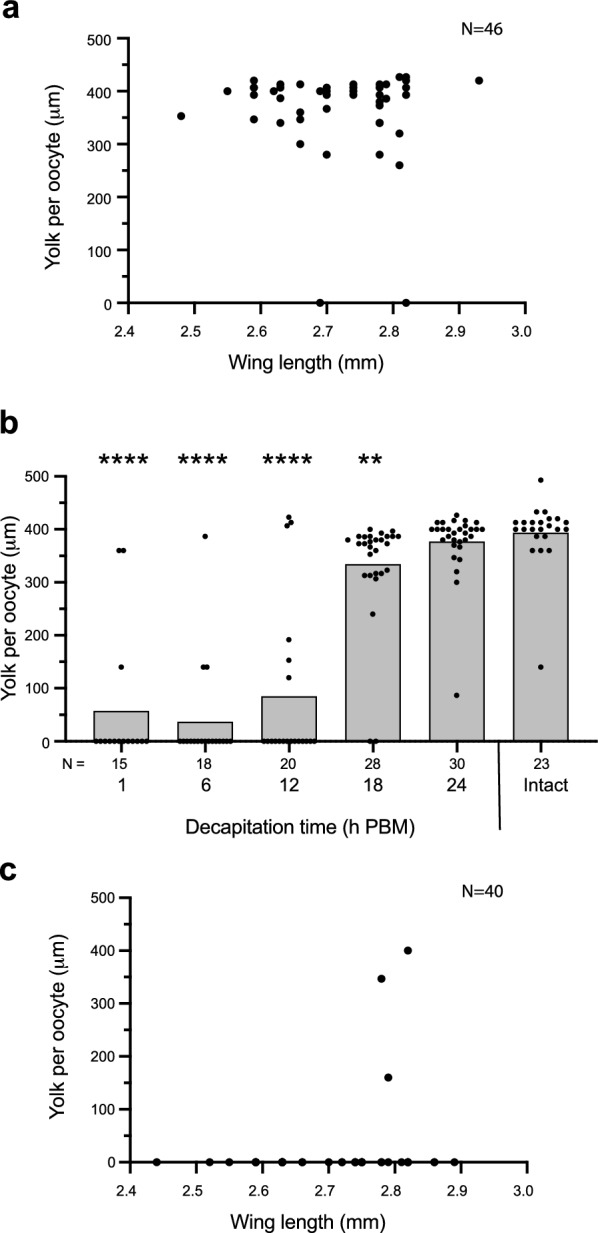
Head-dependent activation of egg development in *An. stephensi.* **a** Yolk deposition per oocyte at 48 h post blood meal (PBM) in relation to body size of intact *An. stephensi* females.* N* in the upper right of the graph indicates the total number of females analyzed. A Spearman rank correlation test detected no significant relationship between yolk per oocyte and body size as measured by wing length (*r* = 0.07; *P* = 0.65). **b** Yolk deposition per oocyte 48 h after a female was blood-fed and decapitated at 1, 6, 12, 18 or 24 h PBM. Yolk deposition into oocytes at 48 h PBM in intact (non-decapitated) females served as the positive control. Bars in each graph show the mean amount of yolk per oocyte, and solid circles show yolk per oocyte for each female examined per treatment. The total number of replicates (females) analyzed per treatment are indicated below the* x*-axis. Statistical significance was determined after assessing homogeneity of variances followed by a Kruskal–Wallis and a post-hoc Dunn’s test. Asterisks above a given bar indicate a given treatment significantly differed from intact females at **P < 0.01 and ****P < 0.0001. **c** Yolk deposition per oocyte at 48 h PBM in relation to body size of *An. stephensi* females that were decapitated at 1 h PBM.* N* in the upper right of the graph indicates the total number of females analyzed. A Spearman rank correlation test detected no significant relationship between yolk per oocyte and body size as measured by wing length (*r* = 0.29; *P* = 0.07)

We first measured the timing of yolk deposition in mated, 4 day PE *An. stephensi* females after blood-feeding to repletion. Yolk per oocyte increased from 1 to 48 h PBM before plateauing (Additional file 1: Figure S1). As a result, eggs were at maximum size at 48 and 60 h PBM, which was followed by most females ovipositing by 72 h PBM. This chronology is very similar to egg maturation by mated, 4 day PE *Ae. aegypti* females that blood-feed to repletion and are maintained under the same conditions [13]. Prior results have reported size-dependent differences in adult fitness traits in *Anopheles* spp. that could affect egg formation [53]. The *An. stephensi* adult females we used in our experiments varied as measured by wing length from approximately 2.5 to 3.0 mm. We thus used a second cohort of 4 day PE females to blood-feed to repletion and examined whether maximum egg size at 48 h PBM correlated with adult female size. Plotting the size of each female against yolk deposited per oocyte indicated it did not (Fig. 2a). Two females in this sample deposited no yolk per oocyte after blood-feeding to repletion, but neither was among the smallest females examined (Fig. 2a). We thus concluded yolk deposition into oocytes by *An. stephensi* females reached a maximum at 48 h PBM and did not vary with adult size under our laboratory rearing conditions.

We therefore next used 4 day PE females with wing lengths that ranged from 2.5 to 3.0 mm to determine when sufficient amounts of head-produced hormones were released in *An. stephensi* to stimulate yolk deposition. This was approached by decapitating females at different times PBM followed by measuring yolk per oocyte at 48 h PBM. While sufficient quantities of head-produced hormones are released by 2 h PBM in *Ae. aegypti* [13], most *An. stephensi* females deposited no yolk into oocytes when decapitated 1, 6 or 12 h PBM (Fig. 2b). In contrast, most females decapitated at 18 or 24 h PBM deposited similar amounts of yolk per oocyte as the intact (non-decapitated) females which served as the positive control (Fig. 2b). Thus, most *An. stephensi* females released sufficient quantities of head-produced hormones to stimulate normal levels of yolk deposition into oocytes later than *Ae. aegypti.* However, these assays also identified variation in the timing of the release of head factor or in responsiveness to blood-feeding, with some *An. stephensi* females decapitated at 1–12 PBM depositing yolk into oocytes and a small percentage of females decapitated 18 or 24 PBM or those that were intact depositing little or no yolk (Fig. 2b). We also conducted assays in which we blood-fed 4 day PE females to repletion and then plotted the size of each individual against yolk deposited per oocyte at 48 h PBM for females decapitated at 1 h PBM. Most individuals, as expected, deposited no yolk into oocytes, which in turn resulted in no significant correlation with body size (Fig. 2c). However, three females in this sample did deposit yolk. Each of these had a wing size of approximately 2.8 mm, which was on the higher end of the size distribution, but several other females with wing sizes of ≥ 2.8 mm deposited no yolk per oocyte (Fig. 2c). Thus, a small percentage of intact *An. stephensi* females deposited less yolk per oocyte than other individuals, while a small percentage of females decapitated at 1 h PBM deposited more yolk per oocyte than other individuals. However, neither of these responses significantly correlated with body size.

### ILP3 and ILP4 activate the vitellogenic phase in *An. stephensi*

**Fig. 3:**
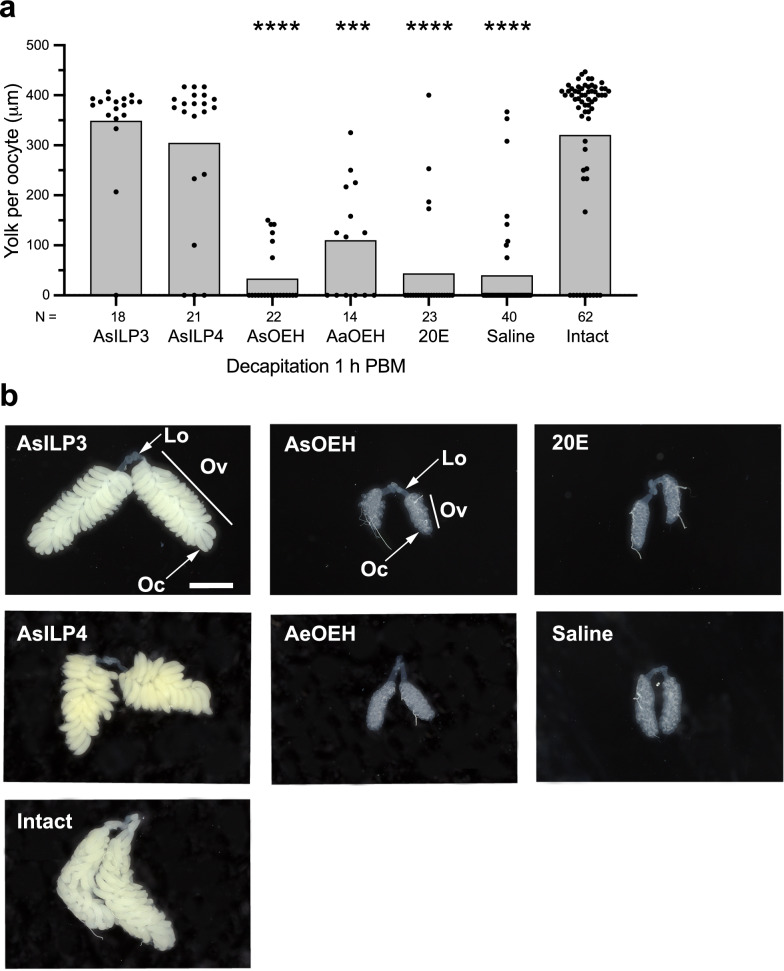
Decapitation followed by hormone treatment variably affects yolk deposition into oocytes. **a** Yolk deposition into oocytes at 48 h PBM after females were blood-fed, decapitated 1 or 12 h post-blood meal (PBM), and injected with AsILP3 (20 pmol), AsILP4 (20 pmol), AsOEH (20 pmol), AaOEH (20 pmol), 20E (1 µmol), or saline (phosphate-buffered saline). Yolk deposition into oocytes at 48 h PBM in intact (non-decapitated) females served as the positive control. Bars show the mean amounts of yolk, and solid circles show yolk per independent replicate for each treatment. Total number of replicates per treatment (females) are indicated below the* x*-axis. Statistical significance for the treatments shown on each graph was determined after assessing homogeneity of variances followed by a Kruskal–Wallis and a post-hoc Dunn’s test. Asterisks above a given bar indicate a given treatment significantly differed from the designated control at ***P < 0.001 and ****P < 0.0001. **b** Light micrograph images showing representative paired ovaries (*Ov*) connected to the lateral oviducts (*Lo*) at 48 h PBM for each treatment in** a**. Each oocyte (*Oc*) per ovary from most females injected with AsILP3 or AsILP4 is enlarged and similar in size to the oocytes in ovaries from intact females due to the uptake of large amounts of yolk. Ovaries from females injected with AsOEH, AeOEH, 20E or saline remain small due to little or no yolk uptake by individual oocytes. The ovary, oocyte and lateral oviduct are labeled in the AsILP3 or AsOEH treatment with the same structures visible in the other treatments. Scale bar (AsILP3 panel): 1 mm; all other images are show at the same magnification. 20E, 20-hydroxyecdysone; AaOEH, *Aedes aegypti* ovary ecdysteroidogenic hormone; ASILP3/4, *Anopheles stephensi* insulin-like peptide 3/4; AsOEH, *Anopheles stephensi* ovary ecdysteroidogenic hormone

Since decapitation at 1 h PBM inhibited most *An. stephensi* females from depositing yolk into oocytes, we tested whether a single 20 pmol dose of two head-produced ILPs or OEH in saline rescued yolk deposition as occurs in *Ae. aegypti* [8, 13]*.* We selected AsILP3, AsILP4 and AsOEH for these assays because prior studies indicate each is produced in mNSCs of anopheline females [29, 32]. We considered the possibility that the AsOEH we produced and purified was defective by injecting a 20-pmol dose of *Ae. aegypti* OEH (AaOEH) into *An. stephensi*; AaOEH is structurally similar and strongly stimulates yolk deposition in *Ae. aegypti* females after decapitation at 1 h PBM [13, 16]. However, *An. stephensi* females injected with AaOEH also deposited little yolk when compared to intact females (Fig. 3a)*.* We also conducted the reciprocal experiment by injecting a 20-pmol dose of AsOEH or AaOEH into *Ae. aegypti* females that were decapitated 1 h PBM. While AaOEH strongly stimulated yolk deposition as found in previously reported studies, AsOEH did not (Additional file 1: Figure S2). This finding indicated that AaOEH was biologically active in *Ae. aegypti* although it had no significant effect on *An. stephensi,* whereas the lack of activity of AsOEH could reflect structural defects or that AsOEH also lacks activity in *Ae. aegypti.* Lastly, we tested whether a single 1 µmol dose of 20E in saline could rescue yolk deposition given that ILPs and OEH interact with 20E to activate the vitellogenic phase in *Ae. aegypti* [8, 20]. The injection of each hormone into females within 1 h PBM showed that AsILP3 and AsILP4 stimulated yolk deposition per oocyte to levels that did not differ from intact females, whereas the AsOEH, 20E or saline treatment did not (Fig. 3a). Light micrographs of ovaries from each of the above treatments showed that a 20-pmol dose of AsILP3 or AsILP4 resulted in ovaries and individual oocytes of similar size to the ovaries and oocytes in intact females (Fig. 3b). In contrast, ovaries from females treated with AsOEH, AaOEH, 20E or saline remained small because these treatments stimulated little or no yolk deposition per oocyte (Fig. 3b).

We next tested two other variables that could potentially account for AsOEH and 20E not stimulating yolk deposition into oocytes in females that were decapitated at 1 h PBM. First, we doubled the amount of AsOEH (40 pmol) or 20E (2 µmol) that was injected into females decapitated at 1 h PBM to test whether dose affected outcomes. However, results showed that doubling the doses used did not alter yolk deposition (Additional file 1: Figure S3). Second, we tested whether ovary responsiveness to AsOEH and 20E is delayed in *An. stephensi* relative to *Ae. aegypti*, since the results shown in Fig. 2 indicated that most females deposited no yolk into oocytes unless decapitated 18 h PBM or later. Females decapitated at 12 h PBM and injected with AsILP3 and AsILP4 deposited as much yolk as intact females, which was the same outcome as occurred when females were decapitated at 1 h PBM (Additional file 1: Figure S4). Females decapitated at 12 h PBM and injected with 20E did deposit more yolk than those decapitated at 1 h PBM (compare Additional file 1 Figure S4 to Fig. 3a). However, the amount of yolk per oocyte was still lower than that in oocytes from intact females (Additional file 1: Figure S4). Females decapitated at 12 h PBM and injected with AsOEH or saline only showed no increase in yolk deposition per oocyte (Additional file 1: Figure S4). These findings thus collectively indicated that AsILP3 and AsILP4 strongly activated the vitellogenic phase and fully rescued yolk deposition in decapitated *An. stephensi* females. In contrast, 20E stimulated some yolk deposition in females that were decapitated at 12 h PBM, suggesting responsiveness to this hormone is delayed relative to ILPs, whereas AsOEH showed no activity in any of the assays we conducted.

### AsILP3 activates upstream processes required for yolk production

**Fig. 4:**
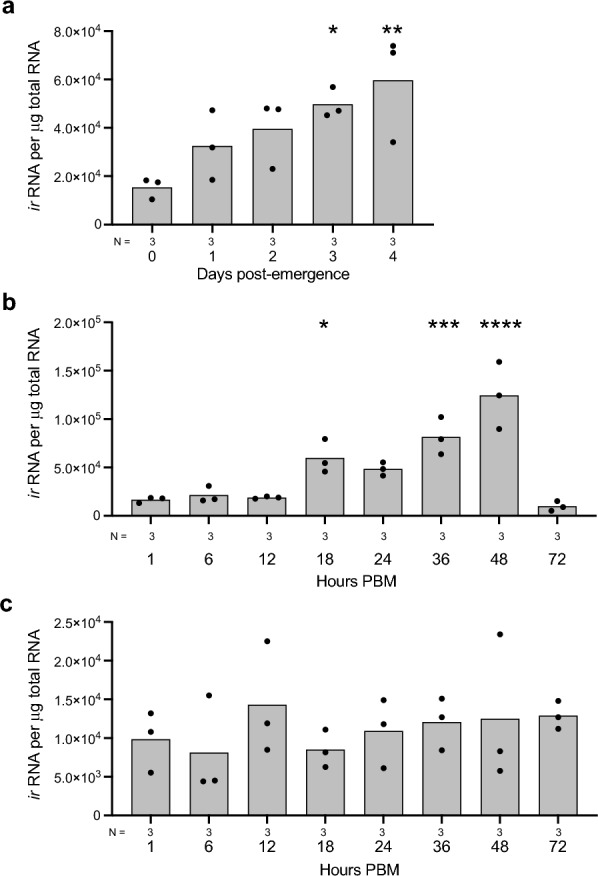
Transcript abundance of the *An. stephensi *insulin receptor (*ir*) during a gonotrophic cycle. **a** Transcript abundance of the *ir* in abdominal pelts containing the ovaries and fat body during the previtellogenic phase. **b** Transcript abundance of the *ir* in 1 ovary pair during the vitellogenic phase. **c** Transcript abundance of the *ir* in abdominal pelts containing the fat body during the vitellogenic phase. In **a**, total RNA was isolated from female abdomens with fat body and ovaries but no gut, at adult emergence (0 days) and 1–4 days post-emergence. The 0 days sample time was designated as the control. **b**, **c** Total RNA was isolated from ovaries or abdomens containing the fat body at 1–72 h PBM. The 1 h PBM time point was designated as the control. Bars in each graph show the mean number of *ir* copies per nanogram of total RNA while solid circles show the number of *ir* copies per independent replicate. The total number of replicates per treatment (abdomens, ovary pairs or abdominal pelts) are indicated below the* x*-axis of each graph. Statistical significance for the treatments shown on each graph was determined after assessment of homogeneity of variances by analysis of variance and a post-hoc Dunn’s test. Asterisks above a given bar in **a** and **b** indicate a given treatment significantly differed from the designated control at *P < 0.05, **P < 0.01, ***P < 0.001 and ****P < 0.0001. No significant differences were detected between the designated control and other time points in **c**. PBM, Post-blood meal;

We assessed three upstream processes strongly associated with IIS and yolk production in *Ae. aegypti* to determine if each process similarly occurred in *An. stephensi.* First, we measured transcript abundance of the IR, which in *Ae. aegypti* binds AaILP3 with high affinity (half-maximal inhibitory concentration [IC_50_] = 5.9 nM) [15]. We began by profiling *An*. *stephensi* *ir* transcript abundance in abdomens of previtellogenic *An. stephensi* females that contained the ovaries and fat body but with gut removed. This was done to establish the baseline transcript abundance levels before a female blood-fed. Since transcript abundance levels of the *ir* in previtellogenic *Ae. aegypti* are low (10^4^ copies/mg total RNA) and similar across tissues [13], we did not separate ovaries from the fat body in previtellogenic *An. stephensi* females. The results showed a similar pattern in *An. stephensi* relative to prior findings in *Ae. aegypti*, with  *ir* copy number in abdomens also being at 10^4^ copies/mg total RNA (Fig. 4a). The results did reveal a significant increase in *ir* transcript abundance in abdomens containing the fat body and ovaries between females on the day of emergence (day 0) versus day 3 or 4 PE (Fig. 4a), but this change was also less than 1 log unit. We then fed 4 day PE females a blood meal and profiled  *ir* transcript abundance in the ovaries and abdominal pelts containing the fat body during the vitellogenic phase. Here, we separated ovaries from the fat body because prior evidence in *Ae. aegypti* indicate   *ir* transcript abundance increases more in the ovaries than in the fat body after a female blood-feeds and releases ILPs produced in mNSCs [13]. The results showed that *ir* transcript abundance in *An. stephensi* increased more than 1 log unit by 48 h PBM when mature eggs had formed before declining by 72 h PBM (Fig. 4b). In contrast, no significant change in *ir* copy number was detected in abdominal pelts containing the fat body from 0 to 72 h PBM (Fig. 4c). Thus, the patterns of *ir* transcript abundance in *An. stephensi* were overall similar to prior findings in *Ae. aegypti.*

**Fig. 5:**
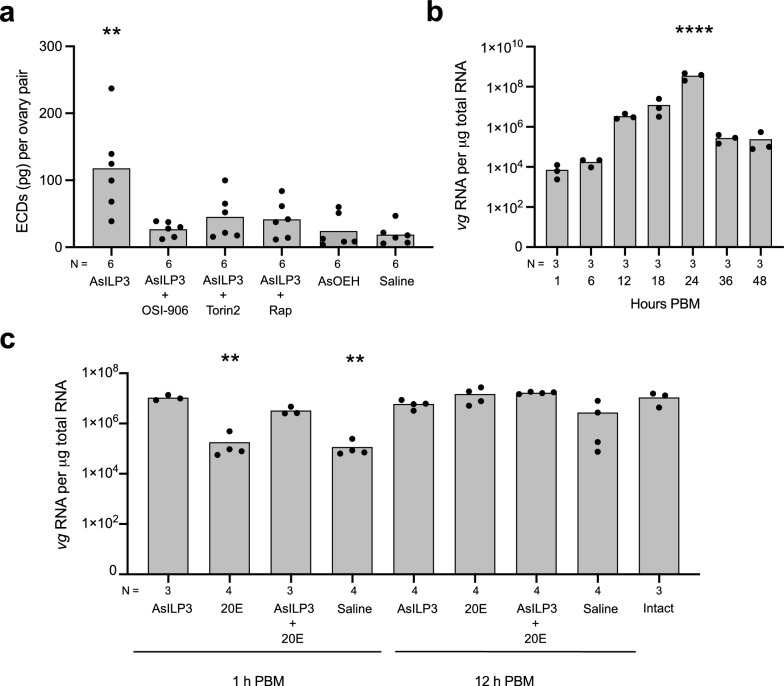
Ecdysteroid production by the ovaries and transcript abundance for the vitellogenin1 (*vg*) gene varies by hormone treatment. **a** ECD production by two ovary pairs treated with AsILP3, AsILP3 + OSI-906, AsILP3 + Torin2, AsILP3 + Rap, AsOEH or saline (which served as the negative control). **b** Transcript abundance for *vg* during the vitellogenic phase from 1 to 48 h PBM. The 1 h PBM time point was designated as the control. **c** Transcript abundance for *vg* at 24 h PBM in females that were decapitated at 1 or 12 h PBM and injected with AsILP3, 20E, AsILP3 + 20E, or saline. Transcript abundance for *vg* at 24 h PBM in intact females served as the positive control. **a-c** Bars show mean amounts of ECD or *vg* while solid circles show each independent replicate per treatment. In **a** ECD per replicate was determined from two ovary pairs collected from two females: thus,* n* values on the* x*-axis indicate the number of ovary pairs analyzed per treatment. **b**, **c** *Vg* per replicate was determined from total RNA that was isolated from a female abdomen in which the ovaries and gut were removed: thus,* N* values under the* x*-axis indicate the number of abdominal pelts analyzed per treatment and the PBM time point. Statistical significance for the treatments shown on each graph was determined after assessing homogeneity of variances followed by a Kruskal–Wallis test (**a**, **b**) or analysis of variance (**c**) and a post-hoc Dunn’s test. Asterisks above a given bar indicate the treatment significantly differed from the control at **P < 0.01 and ****P < 0.0001. 20E, 20-hydroxyecdysone; ASILP3, *Anopheles stephensi* insulin-like peptide 3; AsOEH, *Anopheles stephensi* ovary ecdysteroidogenic hormone; ECD, ecdysteroid; PBM, post-blood meal; RAP, rapamycin

Second, we assessed the requirement for IIS in ECD production by *An. stephensi* ovaries using an established ex vivo assay. For these assays, we focused on AsILP3, since this AsILP3 and AsILP4 were found to be equivalent in terms of stimulating yolk deposition in decapitated females in the preceding assays. We also designated the saline-only treatment as the control group since it was used as the carrier for each hormone. Adding 20 pmol of AsILP3 to ovary cultures in medium containing amino acids significantly increased ECD production when compared to the negative control (Fig. 5a). In contrast, inclusion of OSI-906, which is a potent inhibitor of the IR in *Ae. aegypti* [8, 13], suppressed the stimulatory activity of AsILP3 (Fig. 5a). Inclusion of torin-2 or rapamycin, both of which inhibit TOR kinase activation [8, 25, 52], also suppressed the stimulatory effect of AsILP3, indicating interdependence of IIS and TOR signaling for ECD production (Fig. 5a). Since OEH strongly stimulates ovaries to produce ECD in *Ae. aegypti* [12, 54], we also tested the stimulatory effects of adding 20 pmol of AsOEH to *A. stephensi* ovary cultures. However, our results showed that AsOEH did not stimulate any increase in ECD production (Fig. 5a). These findings were fully consistent with AsILP3 strongly stimulating yolk deposition in *An. stephensi* while AsOEH did not.

Third, we measured the effects of AsILP3 and 20E on transcript abundance of the *vg* gene in *An. stephensi*, which encodes the main protein in yolk. We focused on *vg* transcript abundance because prior studies in *Ae. aegypti* clearly indicate that increased production of this yolk protein during the vitellogenic phase is regulated at the level of transcription and that increased *vg* transcript abundance correlates with increased production of Vg protein [13, 23, 27]. Analysis of blood-feeding 4 day PE females showed that *vg* transcript abundance increased by 24 h PBM to levels that were significantly higher than those at 1 h PBM before declining by 48 h PBM when yolk deposition per oocyte maximized in the preceding assays we conducted (Fig. 5b). We then decapitated females at 1 or 12 h PBM and injected a 20 pmol dose of AsILP3, a 1 mmol dose of 20E, both of these hormones together or saline alone, followed by measurement of *vg* copy number at 24 h PBM. Intact females served as the positive control. We selected these times because AsILP3 had shown yolk-stimulating activity regardless of decapitation time in the preceding assays, whereas 20E only showed activity if decapitation was delayed to 12 h PBM. For females that were decapitated at 1 h PBM, both AsILP3 and AsILP3 + 20E increased *vg* transcript abundance to levels that did not differ from those of the positive control (intact females), whereas the 20E and saline treatments did not (Fig. 5c). For females decapitated at 12 h PBM, all treatments resulted in *vg* copy numbers that did not differ from the positive control, which suggested that sufficient amounts of endogenous hormones were produced to upregulate *vg* expression before females were decapitated (Fig. 5c). Altogether, *vg* copy showed sensitivity to stimulation by AsILP3 or AsILP3 + 20E when females were decapitated at 1 h PBM.

### AsILP3 and 20E only activate the vitellogenic stage in blood-fed females

**Fig. 6:**
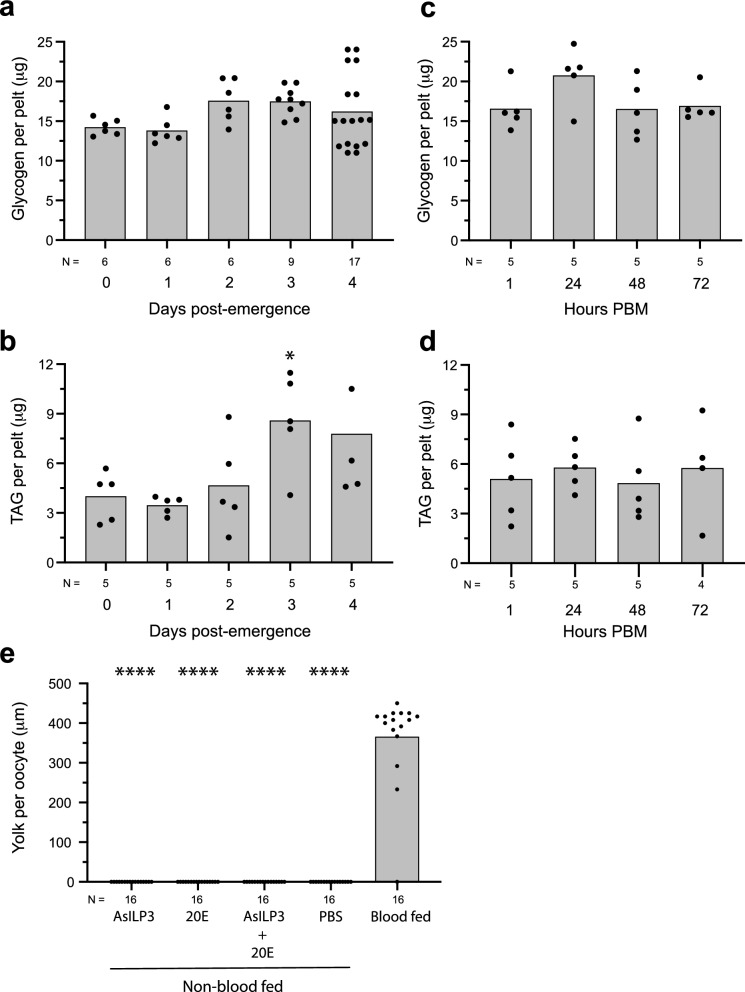
Nutrient stores in the fat body exhibit few changes during a gonotrophic cycle. **a**, **b** Glycogen and TAG stores during the previtellogenic phase. **c**, **d** Glycogen and TAG stores during the vitellogenic phase. **e** Yolk deposition into oocytes 48 h after decapitation of 4 day post-emergence females (non-blood-fed) and injection of AsILP3, 20E, AsILP3 + 20E, or saline (PBS). Intact (non-decapitated), 4 day post-emergence females that were blood-fed served as the positive control. **a**, **b** Glycogen and TAG were determined per female pelt at adult emergence (0 days) and at 1–4 days post-emergence. The 0 day time point was designated as the control. **c**, **d** Glycogen and TAG were determined per female pelt at 1–72 h PBM. **e** Yolk amount per replicate was determined by measuring yolk length (mm) of 3 oocytes per female. Bars in each graph show mean amounts of glycogen, TAG or yolk, and solid circles show each independent replicate per treatment. The total number of replicates per treatment (females) is indicated below the* x*-axis of each graph. Statistical significance for the treatments shown on each graph was determined after assessing homogeneity of variances followed by analysis of variance (**a**–**d**) or a Kruskal–Wallis test (**e**) and a post-hoc Dunn’s test. Asterisks above a bar indicate a given treatment significantly differed from the designated control at *P < 0.05 and ****P < 0.0001. 20E, 20-hydroxyecdysone; ASILP3, *Anopheles stephensi* insulin-like peptide 3; PBM, post-blood meal; PBS, phosphate-buffered saline; TAG, triacylglycerol

Blood-feeding normally activates the vitellogenic phase in *Ae. aegypti,* but previtellogenic females (4 day PE) that have not blood-fed also provision yolk into oocytes if injected with AaILP3 or AaOEH due to the presence of sufficient nutrient reserves in the fat body to do so [42]. This response is also of comparative interest because related species like *Aedes atropalpus* have evolved to be facultatively autogenous: i.e. naturally releasing ILPs and OEH from mNSCs after adult emergence, which stimulates the formation of a first clutch of eggs without blood-feeding, followed by the requirement to blood-feed to produce additional egg clutches [43]. In contrast, autogeny is unknown among anopheline mosquitoes, which could reflect the results from previous studies showing lower nutrient reserves when compared to culicines [36, 37]. Nutrients are primarily stored in the fat body as neutral lipids, such as triacylglycerol (TAG) and glycogen, in *Ae. aegypti* [19]. We thus asked if *An. stephensi* females are similar to or whether they differ from *Ae. aegypti* in terms of being able to increase nutrient stores after adult emergence or to respond to hormones like ILPs and produce eggs in their absence without blood-feeding. We first measured whether previtellogenic *An. stephensi* females increase nutrient stores during the previtellogenic phase when provisioned with sugar. Our results showed that glycogen stores ranged from approximately 14 to 24 mg per pelt in previtellogenic *An. stephensi* females, with no significant change from day 0 to day 4 PE (Fig. 6a). TAG stores modestly but significantly increased by day 3 PE when compared to day 0 (Fig. 6b). Comparing these results to prior measures in *Ae. aegypti* [19] also indicated that glycogen stores were similar but TAG stores were more than fivefold lower. As earlier noted, activation of the vitellogenic phase in *Ae. aegypti* mobilizes nutrient stores at 1–24 h PBM in conjunction with *vg* expression, which is followed by replenishment of nutrient stores under IIS regulation [19]. In contrast, blood-feeding *An. stephensi* females on day 4 PE was followed by no changes in glycogen or TAG stores during the vitellogenic phase (Fig. 6c, d). We then assessed whether nutrient stores in previtellogenic *An. stephensi* females enable females to deposit yolk into oocytes in the absence of blood-feeding, as occurs in *Ae. aegypti* and related culicine species when stimulated with ILPs, OEH and/or 20E. We tested this possibility by injecting 4 day PE females (non-blood-fed) with a 20 pmol dose of AsILP3, a 1 µmol dose of 20E or both hormones, followed by comparison of yolk deposition in females at 48 h to 4 day PE that were blood-fed and examined at 48 h PBM. None of the hormone treatments stimulated any yolk deposition in *An. stephensi* females that did not blood-feed, whereas all blood-fed females produced mature eggs with large amounts of yolk (Fig. 6e). Taken together, these findings indicated *An. stephensi* females greatly differed from *Ae. aegypti* by showing no alterations in nutrient stores during either the previtellogenic phase when sugar-fed or during the vitellogenic phase after blood-feeding. Moreover, *An. stephensi* females also showed no capacity to produce and package yolk into oocytes without blood-feeding.

### Mating increases the number of mature eggs that females produce

**Fig. 7:**
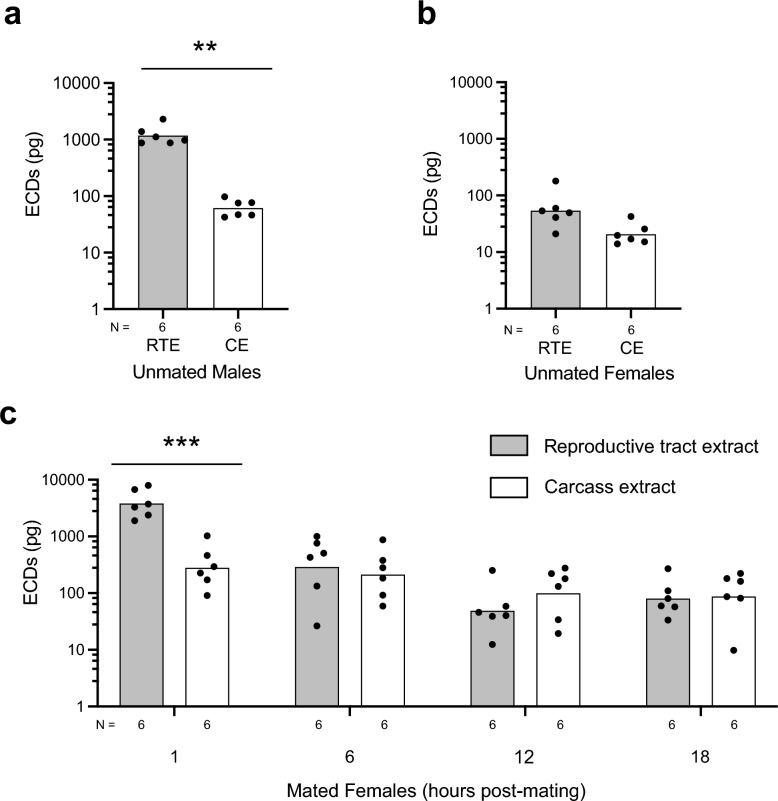
Males transfer ECDs to females during mating. **a** ECDs in RTE and CE prepared from unmated males at 4 day post-emergence. **b** ECDs in RTE and CE prepared from unmated females at 4 day post-emergence. **c** ECDs in the RTE and CE prepared from 4 day post-emergence females 1, 6, 12 and 18 h post-mating. **a**–**c** Bars show mean amounts of ECD per treatment, and solid circles show each independent replicate per treatment. The total number of replicates per treatment is indicated below the* x*-axis of each graph. Each unit of replication was the reproductive tract or carcass extract prepared from a female. Statistical significance was determined after assessing homogeneity of variances followed by two-tailed paired t-tests. Asterisks indicate that the amount of ECD in RTE differed significantly from that in CE in unmated males at **P < 0.01, whereas no difference was detected between RTE and CE in unmated females. **c** Asterisks indicate the amount of ECD in RTE and CE differed between mated females at 1 h post-mating at ***P < 0.001, but did not differ at 6, 12 or 18 h post-mating. CE, Carcass extract; ECDs, ecdysteroids; RTE, reproductive tract extract

As earlier noted, males transfer ECDs in accessory gland secretions to females in *An. stephensi* and select other species in the genus *Anopheles* [48]. Male transfer of ECDs to females in *An. gambiae* also induces mating refractoriness, which is associated with mated females producing more mature eggs after blood-feeding than unmated females [47, 48, 55]. In contrast, how ECD levels in the reproductive tract change after mating and whether ECD transfer by males affects the processes that regulate yolk production and packaging into oocytes after blood-feeding has not been studied. Given this lack of knowledge, we first measured ECD in the reproductive tract extracts from 4 day PE unmated males, 4 day PE unmated females and 4 day PE females at 1, 6, 12 or 18 h after mating with a male who had not previously mated. We also measured ECD in carcass extracts for each of these treatments, which provided a measure for ECD in hemolymph and other tissues. In unmated males, ECD was significantly more abundant in reproductive tract extracts (≥ 1000 pg) than in the carcass extracts (≤ 100 pg), a result that is consistent with those from prior studies, indicating that *An. stephensi* males produce ECD in their reproductive tract (Fig. 7a). In unmated females, the amount of ECD detected in reproductive tract and carcass extracts was similarly low (≤ 100 pg) and did not differ from each other (Fig. 7b). In contrast, ECD increased to more than 1000 pg in reproductive tract extracts of females 1 h after mating with a male (Fig. 7c). The amount of ECD in the reproductive tract extracts of females at 1 h post-mating was also significantly higher than the level of ECD that was detected in carcass extracts at 1 h (Fig. 7c). This large increase in reproductive tract extracts at 1 h post-mating was consistent with the transfer of ECD from males, but was not consistent with any increased production of ECD by the reproductive tract itself given prior measures indicating mosquito ovaries require several hours to increase ECD production after blood-feeding [8, 16–18]. In contrast, ECD detected in the reproductive extracts of mated females declined and did not differ from ECD detected in carcass extracts at 6, 12 and 18 h post-mating (Fig. 7c). This finding indicated that ECD transferred from males does not persist in the reproductive tract of females.

Assessment of yolk deposition into oocytes at 48 h PBM indicated that unmated females deposited large amounts of yolk per oocyte but that the values overall were significantly lower than the amount of yolk per oocyte that was deposited by mated females (Additional file 1: Figure S5a). Within these samples, a larger number of unmated than mated females deposited no yolk into oocytes. However, omitting all unmated or mated females that deposited no yolk into oocytes and re-analyzing the data still indicated that unmated females deposited less yolk per oocyte than mated females (*t* = 2.6,* df* = 47; *P* = 0.011). We hypothesized that mated females deposited more yolk per oocyte than unmated females because ECD transfer from males resulted in ovaries producing more ECD after blood-feeding. However, measurement of ECD production showed no differences between ovaries from mated and unmated females, which rose to a similar maximum at 18 PBM before declining to similar levels at 48 h PBM (Additional file 1: Figure S5b). We also assessed whether ECD transfer from males resulted in higher *vg* transcript abundance levels in the fat body of mated versus unmated females. Our results showed that *vg* transcript abundance similarly rose at 12–24 h PBM in unmated and mated females before declining to baseline levels by 48 h PBM (Additional file 1: Figure S5c). Transcript abundance was slightly but significantly higher in unmated than mated females at 12 h PBM, but the reverse was the case at 24 h PBM. Thus, mated *An. stephensi* females packaged more yolk per oocyte than unmated females, but this increase was not associated with ovaries from mated females producing more ECD or differences in *vg* transcript abundance in the fat body between mated and unmated females.

### *Anopheles stephensi* females mate after consuming a first blood meal but only lay eggs after a second blood meal

**Fig. 8:**
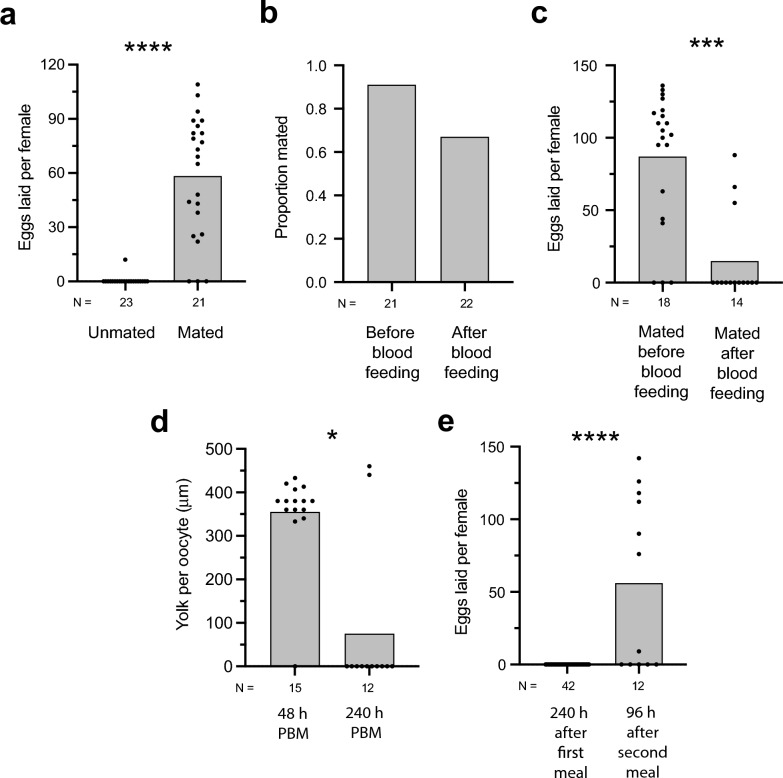
Mating before and after blood-feeding differentially affects egg-laying. **a** Number of eggs laid by unmated and mated females at 96 h PBM. Unmated or mated females were 4 days post-emergence (PE) when blood-fed to repletion. Bars in each graph show mean values, and solid circles show the number of females analyzed per treatment. The total number of females examined per treatment is indicated below the* x*-axis. Statistical significance was determined after assessing homogeneity of variances, followed by a two-tailed Mann–Whitney test. The asterisks above each graph indicate unmated and mated females significantly differed at ****P < 0.0001. **b** Proportion of females that mated if held with males before or after blood-feeding to repletion at 4 days PE. Total number of females examined per treatment is indicated below the* x*-axis. A Fisher’s exact test did not detect a significant difference between the two treatments at *P* = 0.07. **c** Number of eggs laid by females that mated before or after blood-feeding to repletion. The total number of females examined per treatment is indicated below the* x*-axis. Statistical significance was determined as described in **a** (***P < 0.001). **d** Yolk deposition per oocyte 48 h and 10 days (= 240 h) after unmated 4 day PE females blood-fed to repletion followed by mating. Bars show mean values, and solid circles show the number of females analyzed per treatment. Total number of females analyzed per treatment is indicated below the* x*-axis. Statistical significance was determined after assessing homogeneity of variances followed by a two-tailed Mann–Whitney test. The single asterisk indicates the treatments significantly differed at *P = 0.03. **e** Number of eggs laid after unmated, 4 day PE females blood-fed to repletion followed by mating. The left bar shows the number of eggs laid 240 h (= 10 days) after blood-feeding. The right bar shows unmated females that consumed a blood meal at 4 days PE, followed by mating and reabsorption of eggs by 10 days PBM. Females then consumed a second blood meal to repletion. The number of eggs laid was then determined 96 h after consumption of the second blood meal. Bars show mean values, and solid circles show the number of females analyzed per treatment. The total number of females examined per treatment is indicated below the* x*-axis. Statistical significance was determined after assessing homogeneity of variances followed by a two-tailed Mann–Whitney test. Asterisks indicate the treatments significantly differed at ****P < 0.0001. PBM, post-blood meal

In addition to mated *An. gambiae* females producing larger eggs, prior results indicate that mated *An. gambiae* females usually lay eggs after consuming a first blood meal while unmated females do not [55–57]. We assessed whether *An. stephensi* behaved similarly by placing one cohort of newly emerged, unmated females 1–3 days PE with males, which results in mating, while holding another cohort of newly emerged unmated females without males for the same period of time. Dissection of females at day 4 PE indicated that all females with males 1–3 days PE were mated, as evidenced by sperm in the spermatheca, while all females held without males were unmated. Four day PE mated and unmated females were then blood-fed to repletion and provided with oviposition substrates at 96 h PBM to determine how many eggs were laid. The results showed that most mated females laid eggs while most unmated females did not, as previously found for *An. gambiae* (Fig. 8a). We next confined a cohort of females with males 1–3 days PE, followed by allowing 4 day PE females to blood-feed to repletion. These females were then provided an oviposition substrate at 96 h PBM. A second cohort of unmated, 4 day PE females were also allowed to blood-feed to repletion but were then placed in cages with excess males for 72 h PBM, followed by provision of an oviposition substrate at 96 h PBM. We then compared the proportion of females in each cohort that were mated by inspection of the spermatheca and counted the number of eggs each female laid. More than 90% of females held with males before blood-feeding mated, while 70% of females held with males after blood-feeding did so; this difference was not significant (Fig. 8b). In contrast, while almost all females that mated before blood-feeding laid eggs, most females that mated after blood-feeding did not (Fig. 8c). Dissection of the females that mated after blood-feeding but laid no eggs showed that the ovaries of each contained mature eggs. We also compared the number of eggs laid by mated 4 day PE females after consuming a blood meal to repletion to unmated 4 day PE females that after blood-feeding were injected with 20E at 48 h PBM to simulate ecdysteroid transfer from a male. While most mated females laid eggs, most unmated females laid no eggs after injection of 20E or saline which served as a negative control (Additional file 1: Figure S6). These experiments thus collectively indicated: (1) 4 day PE *An. stephensi* females that mated before consuming a blood meal to repletion laid eggs by 96 h PBM; (2) most unmated 4 day PE females also blood-fed and mated with males after blood-feeding; (3) most 4 day PE females that mated after consuming a blood meal matured but laid no eggs when given the opportunity to do so at 96 h PBM; and (4) unmated 4 day PE females that were injected with 20E 48 h PBM also laid no eggs.

Given preceding outcomes, we next blood fed a third cohort of unmated, 4 day PE females to repletion followed by placing them in cages with excess males for 72 h as above. We dissected 32 of these individuals at 48 h PBM which showed that most contained oocytes with more than 300 mm of yolk (Fig. 8d). In the preceding experiment, most females that mated after blood-feeding laid no eggs at 96 h PBM (see above). We therefore held the remainder of the females (*n* = 42) in this third cohort for 10 days (= 240 h) PBM before providing an oviposition substrate. Again, none laid any eggs (Fig. 8e). However, dissection of 12 of these females on day 11 PBM showed that most contained no oocytes with yolk (Fig. 8d). Since most females contained oocytes with yolk at 48 h PBM, the absence of yolk at 10 days PBM indicated these resources were reabsorbed. That females reabsorbed eggs also led us to test whether the remaining females (*n* = 30) of the third cohort would consume a second blood meal. Twelve blood fed to repletion while 18 did not. Seven of the females that blood fed to repletion laid eggs when given an oviposition substrate at 96 h PBM which was in stark contrast to no females that mated after consuming a first blood meal doing so (Fig. 8e). Dissecting the five females that laid no eggs after a second blood showed that 4 contained no oocytes with yolk while one contained oocytes with an average of 440 µm of yolk per oocyte. Thus, *An. stephensi* females that mated after consuming a blood meal matured but usually laid no eggs that were reabsorbed by 10 days PBM. However, a majority of these females that consumed a second blood meal did lay eggs.

## Discussion

We introduced this study by summarizing four areas in which the regulation of gonotrophic cycles could differ between culicine mosquitoes like *Ae. aegypti* and anopheline mosquitoes like *An. stephensi*. The first area is if head-produced hormones activate the vitellogenic phase, which is well-established in *Ae. aegypti* but unstudied in anophelines. As noted in earlier studies, time-course decapitation assays experimentally demonstrated that *Ae. aegypti* females release sufficient quantities of head-produced hormones by 2 h PBM to stimulate normal yolk deposition into oocytes by 48 h PBM. These hormones were subsequently identified as ILPs and OEH that are produced in mNSCs [8, 12, 13, 27]. We report in this study that *An. stephensi* females, like *Ae. aegypti,* also produce mature eggs by 48 h PBM. Unlike *Ae. aegypti*, however, time-course decapitation assays indicated that sufficient quantities of head-produced hormones to stimulate normal egg development are not released until 18 h PBM in most *An. stephensi* females. However, our results also show that some *An. stephensi* females decapitated 1, 6 or 12 h PBM do deposit yolk into oocytes earlier than 18 h, including some that deposit as much yolk per oocyte as intact females which served as the positive control for these experiments. These findings overall indicate most *An. stephensi* females release sufficient amounts of head-produced hormones to activate the vitellogenic phase later than *Ae. aegypti*, but also suggest that release times are more variable. A similar pattern was earlier suggested by experiments conducted in *Anopheles albimanus* [58], which suggests that *Anopheles* spp. generally may vary more in the timing of head-factor release than *Ae. aegypti*.

This variation is why in the second part of the study we decapitated females at 1 or 12 h PBM to test whether ILPs and OEH exhibit the same stimulatory activity in *An. stephensi* as previously determined for *Ae. aegypti.* The results we report here identify AsILP3 and AsILP4 as head-produced hormones that rescue the vitellogenic phase in *An. stephensi* after blood-feeding and decapitation*,* while AsOEH and AaOEH do not*.* We also tested the rescue activity of 20E because: (1) prior findings in *Ae. aegypti* and *An. stephensi* indicate ILPs stimulate the ovaries to produce ECD [8, 13, 33]; and (2) ILPs and 20E together more strongly stimulate the fat body in *Ae. aegypti* to produce yolk proteins like Vg and other physiological processes during the vitellogenic phase than either hormone does alone [13, 19, 25, 26, 59]. A differential responsiveness of *An. stephensi* to injection of 20E alone is suggested by our finding that decapitated females deposit little or no yolk into oocytes if treated immediately after decapitation at 1 h PBM but that they do deposit some yolk if treated with 20E after decapitation at 12 h PBM. In contrast, our results do not indicate co-injection of ILPs and 20E increases yolk per oocyte at 48 h PBM over injection of AsILP3 alone.

The modest PE increase in *ir* transcript abundance in the abdomens of previtellogenic *An. stephensi* is similar to that in *Ae. aegypti* [13] and consistent with females becoming competent to blood-feed. Further increases in *ir* copy numbers in ovaries after blood-feeding is also similar to those observed in *Ae. aegypti* where upregulated expression is associated with ILP-induced synthesis of ECDs [8, 13]. Further increases 36–48 h PBM in *An. stephensi* suggest that additional *ir* copies may be important to complete yolk packaging into oocytes and chorion formation and activate development of secondary follicles. That *ir* copy number does not increase during the vitellogenic phase in the fat body is similar to the situation in *Ae. aegypti* and consistent with the conclusion that receptor abundance is sufficient to produce Vg and other yolk components. The experiments we conducted in this study also indicate that AsILP3 strongly stimulates ECD production by the ovaries and upregulated expression of *vg* in the fat body. In contrast, 20E alone has no effect on *vg* transcript abundance while AsILP3 + 20E does not increase *vg* expression more than AsILP3 alone. These outcomes thus collectively identify ILPs as the primary hormones that activate the vitellogenic phase in *An. stephensi*.

We standardized our hormone injection assays by only using mated 4 day PE females that blood-fed once to repletion. We also carefully controlled rearing densities and feeding regimens for larvae, which resulted in adult females of similar size (wing length: 2.5–3.0 mm) since prior studies indicate anopheline larvae that develop in overcrowded conditions suffer nutritional stress that results in higher mortality and smaller adults [53, 60, 61]. Prior studies of anophelines note that small, adult females from overcrowded conditions can also emerge in a pre-gravid state that for the first gonotrophic cycle requires consumption of two or more blood meals to produce and lay eggs [62–66]. In contrast, it has also been suggested pre-gravid states may be more common in *An. gambiae* and *An. funestus* than in other *Anopheles* spp. including *An. stephensi* [37, 53]. The larval rearing conditions used in this study minimized nutrient stress and subsequently the development of small *An. stephensi* females. However, the size of females used in the study were not identical, which is why we examined whether this variable explains why some intact females deposited less yolk than others, or why a few females decapitated 1 h PBM deposited yolk into oocytes when most decapitated females did not. However, our results show that female size does not correlate with yolk deposition per oocyte in either intact females or females that were decapitated at 1 h PBM. We thus conclude the variation in when sufficient quantities of ILPs are released from mNSCs after blood-feeding to stimulate formation of mature eggs is not predicted by body size. We also note that one study detected the expression of AsILP family members in other body regions besides the head [32], possibly suggesting that the release of AsILP3 and AsILP4 in sufficient quantities to activate the vitellogenic phase occurs after blood-feeding from other cells besides mNSCs. Several peptide hormones in mosquitoes are produced by neurosecretory cells in ganglia of the ventral nerve that extend to the thorax and abdomen [11, 32, 67]. However, future studies will be needed to determine if the ventral nerve chord is a source of ILPs that can activate the vitellogenic phase in *An. stephensi.*

We focused the third part of this study on measuring nutrient stores in *An. stephensi* females. Prior experiments conducted with *An. gambiae* show that glycogen and lipid stores are higher in previtellogenic females with access to sugar than in females with access to only water [68]. In contrast, we focused our experiments on how glycogen and TAG stores in previtellogenic *An. stephensi* compared to those in *Ae. aegypti*. We show that glycogen stores in the fat body of previtellogenic *An. stephensi* are very similar to those in *Ae. aegypti,* whereas TAG stores are much lower, which is consistent with findings indicating total nutrient stores are lower in previtellogenic anopheline mosquitoes than in *Ae. aegypti* and select other culicines [36]. Unlike *Ae. aegypti* [11], previtellogenic *An. stephensi* also show little capacity to increase nutrient stores in response to sugar consumption, while blood-feeding neither mobilizes nor increases nutrient stores*.* These results together with those from prior studies collectively suggest that anophelines cannot accumulate nutrient reserves as large as *Ae. aegypti* and other culicines [36, 37, 53]. More limited nutrient reserves also likely affect other life history traits, including the ability to produce eggs without blood-feeding (autogeny), which has independently evolved several times in the Culicinae but is unknown in the Anophelinae [6, 22, 42, 43, 69, 70]. A number of culicine mosquitoes have also evolved to provision eggs with sufficient nutrient stores to survive long periods via diapause or quiescence [39–41], while limited nutrient stores likely contribute to anophelines being unknown to lay diapausing or quiescent eggs. In contrast, prior studies do suggest that adult *An. gambiae* have evolved the capacity for long-range migration while adult *An. coluzzi* show evidence for long-term survival during summer dry seasons via aestivation [71, 72]. However, the physiological processes regulating these responses and associated metabolic costs are unknown.

In the fourth part of the study, we measured ECDs in the adult reproductive tracts of *An. stephensi* males and females. These results corroborate earlier results reporting a subset of species in the genus *Anopheles*, including *An. stephensi*, transfer ECD to females during mating in accessory gland secretions that form a mating plug [46, 48, 57, 73]. However, our results also indicate that these transferred ECDs rapidly diminish in the reproductive tracts of *An. stephensi* females 6–12 h post-mating, which has not previously been reported. Our results also indicate that mated *A. stephensi* females produce eggs containing modestly but significantly more yolk than unmated females. Recent studies in *An. gambiae* indicate that males preferentially transfer a modified form of 20E to females, which suppresses remating and promotes increased egg formation and oviposition by modulating the expression of other factors and signaling processes [74]. In contrast, how these factors stimulate females to deposit more yolk per oocyte than unmated females is unknown. We reasoned that increased ECD production by the ovaries or *vg* expression by the fat body could potentially contribute to mated females packaging more yolk per oocyte than unmated females in *An. stephensi*, but the results we report do not support this. In contrast, our results do identify ILPs as more important than ECDs in terms of activating the vitellogenic phase in *An. stephensi.* If this is the case for anopheline mosquitoes in general, then the mechanisms underlying how ECDs transferred from males to females promote egg formation after blood-feeding is potentially through effects on ILP release or IIS rather than on female-produced ECDs.

Our finding that unmated *An. stephensi* females mature but do not lay eggs after blood-feeding is consistent with the results of prior studies on *An. gambiae.* We thus also examined whether unmated *An. stephensi* females will mate after blood-feeding and stimulate egg-laying as usually occurs when females mate before consuming a blood meal to repletion. We show that most unmated 4 day PE females will mate after consuming a first blood meal to repletion but that mating after blood-feeding results in almost no females laying eggs when tested at 96 h and 10 days PBM. Our finding that *An. stephensi* females mate after blood-feeding is consistent with mesocosm studies that used indirect methods to conclude that some *An. gambiae* females mate after blood-feeding although most individuals mate before blood-feeding [68]. Results from prior studies indicate that some *Aedes* spp. will also mate after blood-feeding [75]. However, our finding that most *An. stephensi* females that mate after blood-feeding do not oviposit contrasts with results in *Ae. aegypti* which do lay eggs [75]. It is unclear from studies in the literature whether anopheline mosquitoes lay eggs if they mate after blood-feeding, but the authors of earlier studies report that 20E injection into unmated *An. gambiae* females 48 h after blood-feeding stimulates a significant increase in the number of females that lay eggs [57]. However, this result does not include an indication of how many eggs females lay [57], and contrasts with other studies reporting that accessary gland secretions, which are the source of transferred ECDs [45], do not stimulate oviposition in *An. gambiae* [55, 56]. Whether these outcomes reflect different experimental designs, culture conditions or other factors will require additional studies. However, unlike previously conducted studies, our results indicate that 4 day PE *An. stephensi* females which mate after a first full blood meal reabsorb eggs by 10 days PBM. We also show that many of these females will consume a second blood meal to repletion, which is followed by the oviposition of eggs. Thus, while almost no *An. stephensi* in our study laid eggs following mating after a first blood meal, a majority of these individuals did lay eggs after consuming a second blood meal. These findings thus suggest mating after blood-feeding results in a change in state that enables females to lay eggs but only if they reabsorb the first clutch of eggs and consume a second blood meal that stimulates development of a second clutch of eggs. This response in effect also results in mating needing to occur before a blood meal for *An. stephensi* females to lay eggs. Additional experiments are needed to determine the mechanisms underlying this response in *An. stephensi* and if the pattern we report in this study occurs in other anophelines.

## Conclusions

Blood-feeding followed by decapitation assays indicated that most *An. stephensi* females release sufficient quantities of head-produced hormones to stimulate yolk deposition into oocytes later than previously reported for *Ae. aegypti.* AsILP3 and AsILP4, which are produced in brain mNSCs, rescued yolk deposition when injected into females that were decapitated at 1 and 12 h PBM. In contrast, AsOEH and AaOEH stimulated little yolk deposition when injected into decapitated females. Consistent with rescuing yolk deposition, AsILP3 stimulated ECD production by ovaries and increased *vg* levels in the fat body. Glycogen stores in the fat body of *An. stephensi* females were similar to previously measured glycogen stores in *Ae. aegypti*, but TAG stores in *An. stephensi* were much lower. Glycogen and TAG stores were not mobilized after *An. stephensi* females blood-fed, while injection of AsILP3 or 20E did not stimulate any yolk deposition into oocytes if injected into previtellogenic (non-blood-fed) females. Male *An. stephensi* transferred ECD to the reproductive tracts of females during mating, which was associated with increased yolk deposition into oocytes after blood-feeding and oviposition of more eggs when compared to unmated females. In contrast, mated and unmated females exhibited few differences in ECD production by the ovaries or in *vg* copy number in the fat body. While unmated *An. stephensi* females matured eggs after blood-feeding, most did not lay eggs. Follow-up assays further showed that neither mating after blood-feeding nor injection of 20E after unmated females blood-fed stimulated oviposition after a first blood meal. However, some *An. stephensi* females that mated after a first blood meal did oviposit eggs after consuming a second blood meal.

## Supplementary Information

**Additional file 1: Figure S1.**  Yolk deposition per oocyte at 1-60 h PBM by *An. stephensi* females.** Figure S2.** Yolk deposition into oocytes at 48 h PBM after *Ae. aegypti* females were blood fed, decapitated 1 h PBM, and injected with AsOEH (20 pmol), AaOEH (20 pmol), or Saline.** Figure S3.** Yolk deposition into oocytes at 48 h PBM after An. stephensi females were blood fed decapitated 1 h PBM, and injected with AsOEH (40 pmol), 20E (2 *u*mol), or Saline.** Figure S4**. Yolk deposition into oocytes at 48 h PBM after *An. stephensi* females were blood fed, decapitated at 12 PBM, and injected with AsILP3 (20 pmol), AsOEH (20 pmol), AaOEH (20 pmol), 20E (1 µmol), or Saline.** Figure S5.** Mated *An. stephensi* females deposit more yolk into oocytes but show no difference in ECD production by the ovaries or *vg* transcript abundance when compared to unmated females.** Figure S6.** Number of eggs laid by mated *An. stephensi* females after blood feeding to repletion versus unmated females that blood fed to repletion that were injected with Saline (negative control) or 20E. 

## Data Availability

Data supporting the main conclusions of this study are included in the manuscript.

## Abbreviations

20E: 20-Hydroxyecdysone
AaOEH: *Aedes aegypti* ovary ecdysteroidogenic hormone
AsILP3: *Anopheles stephensi* insulin-like peptide 3
AsILP4: *Anopheles stephensi* insulin-like peptide 4
*Asir*: *Anopheles stephensi* insulin receptor mRNA
AsOEH: *Anopheles stephensi* ovary ecdysteroidogenic hormone
*Asvg*: *Anopheles stephensi* vitellogenin1 mRNA
ECD: Ecdysteroid
EIA: Enzyme-linked immunoassay
IIS: Insulin/insulin-like growth factor signaling
ILP: Insulin-like peptide
IR: Insulin receptor
JH: Juvenile hormone
mNSC: Medial neurosecretory cell
OEH: Ovary ecdysteroidogenic hormone
OEHR: OEH receptor
PBM: Post-blood meal
PE: Post-emergence
RT: Reproductive tract
TOR: Target of rapamycin
Vg: Vitellogenin
