# Supplementary material for: Insulin-like peptides activate egg formation in the Asian malaria mosquito Anopheles stephensi
Source: Parasit Vectors. 2025 Oct 7;18:399. doi: 10.1186/s13071-025-07036-y (PMC12506429; doi:10.1186/s13071-025-07036-y)
Supplement: Supplementary file 1 — Additional file 1: Figure S1. Yolk deposition per oocyte at 1-60 h PBM by An. stephensi females. Figure S2. Yolk deposition into oocytes at 48 h PBM after Ae. aegypti females were blood fed, decapitated 1 h PBM, and injected with AsOEH (20 pmol), AaOEH (20 pmol), or Saline. Figure S3. Yolk deposition into oocytes at 48 h PBM after An. stephensi females were blood fed decapitated 1 h PBM, and injected with AsOEH (40 pmol), 20E (2 umol), or Saline. Figure S4. Yolk deposition into oocytes at 48 h PBM after An. stephensi females were blood fed, decapitated at 12 PBM, and injected with AsILP3 (20 pmol), AsOEH (20 pmol), AaOEH (20 pmol), 20E (1 µmol), or Saline. Figure S5. Mated An. stephensi females deposit more yolk into oocytes but show no difference in ECD production by the ovaries or vg transcript abundance when compared to unmated females. Figure S6. Number of eggs laid by mated An. stephensi females after blood feeding to repletion versus unmated females that blood fed to repletion that were injected with Saline (negative control) or 20E. [file 13071_2025_7036_MOESM1_ESM.pdf]

Additional file 1 for: Insulin-like peptides activate egg formation in the Asian malaria mosquito *Anopheles stephensi*

Benjamin L. Phipps, Mark R. Brown, and Michael R. Strand

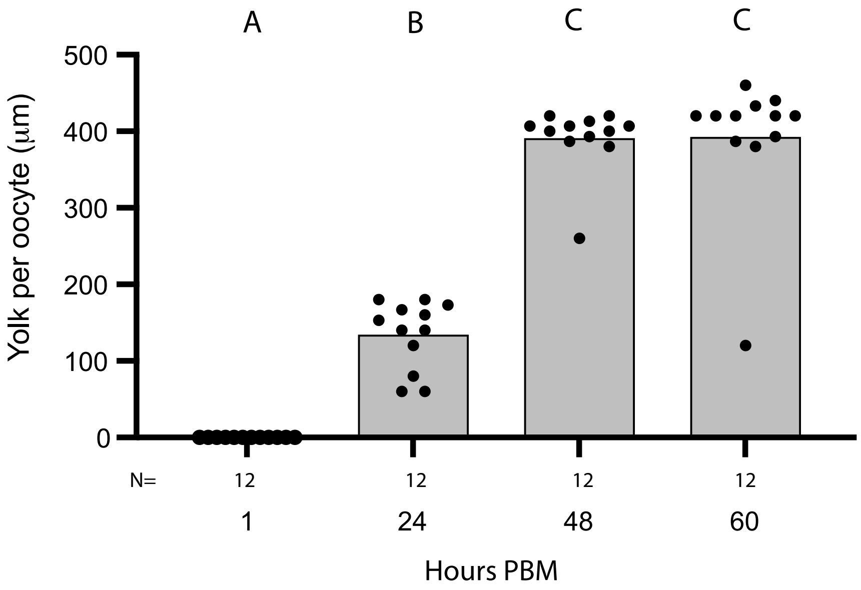

Fig. S1 Yolk deposition per oocyte at 1-60 h PBM by *An. stephensi* females. Bars in each graph show mean amounts of yolk per oocyte while solid circles show yolk per oocyte for each female examined per time point. Total number of replicates (females) for each time point is indicated below each bar on the x axis. Statistical significance for each time point was determined after assessing homogeneity of variances followed by ANOVA and a post-hoc Tukey's multiple comparison test. Different letters above a given bar indicate yolk per oocyte significantly differed ($p \leq 0.05$).

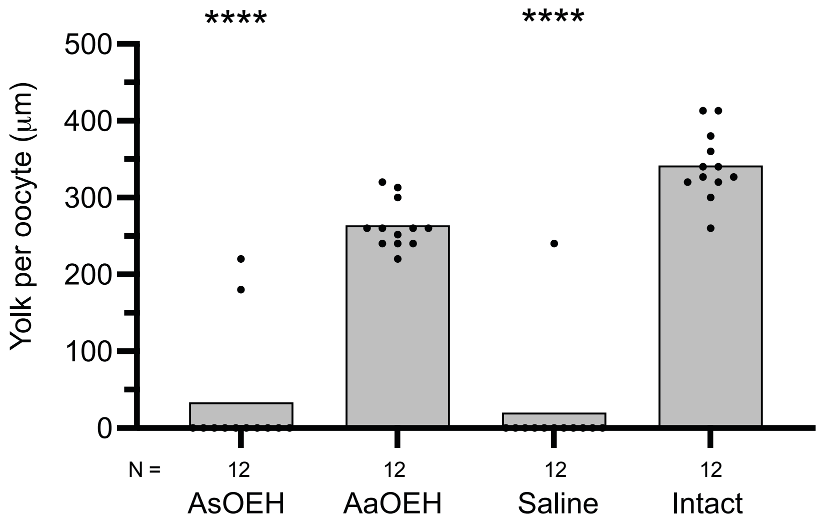

Fig S2 Yolk deposition into oocytes at 48 h PBM after *Ae. aegypti* females were blood fed, decapitated 1 h PBM, and injected with AsOEH (20 pmol), AaOEH (20 pmol), or Saline. Intact (non-decapitated) females served as the positive control. Bars in each graph show mean amounts of yolk per oocyte while solid circles show yolk per independent replicate for each treatment. Total number of replicates (females) per treatment are indicated below the x axis of each bar. Statistical significance for the treatments shown on each graph was determined after assessing homogeneity of variances followed by a Kruskal-Wallis and a post-hoc Dunn's test. Asterisks above a given bar indicates each treatment significantly differed from the designated control (Intact females): $p<0.0001$ (****).

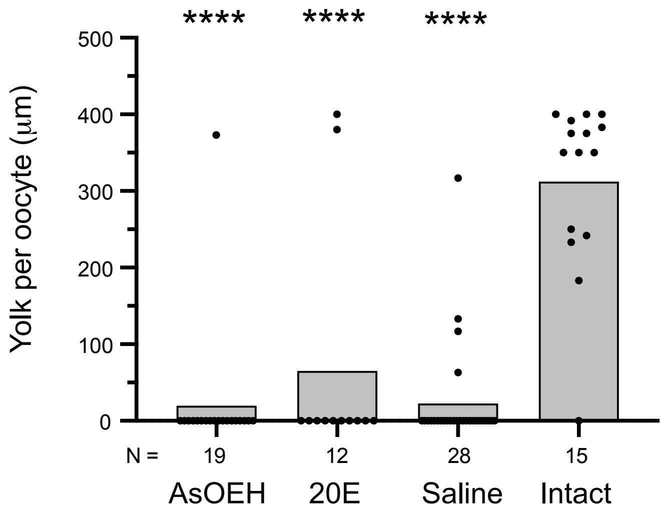

Fig. S3 Yolk deposition into oocytes at 48 h PBM after *An. stephensi* females were blood fed, decapitated 1 h PBM, and injected with AsOEH (40 pmol), 20E (2 μmol), or Saline. Intact (non-decapitated) females served as the positive control. Bars in each graph show mean amounts of yolk per oocyte while solid circles show yolk per independent replicate for each treatment. Total number of replicates (females) per treatment are indicated below the x axis of each bar. Statistical significance for the treatments shown on each graph was determined after assessing homogeneity of variances followed by a Kruskal-Wallis and a post-hoc Dunn's test. Asterisks above a given bar indicates each treatment significantly differed from the designated control (Intact females): $p<0.0001$ (****).

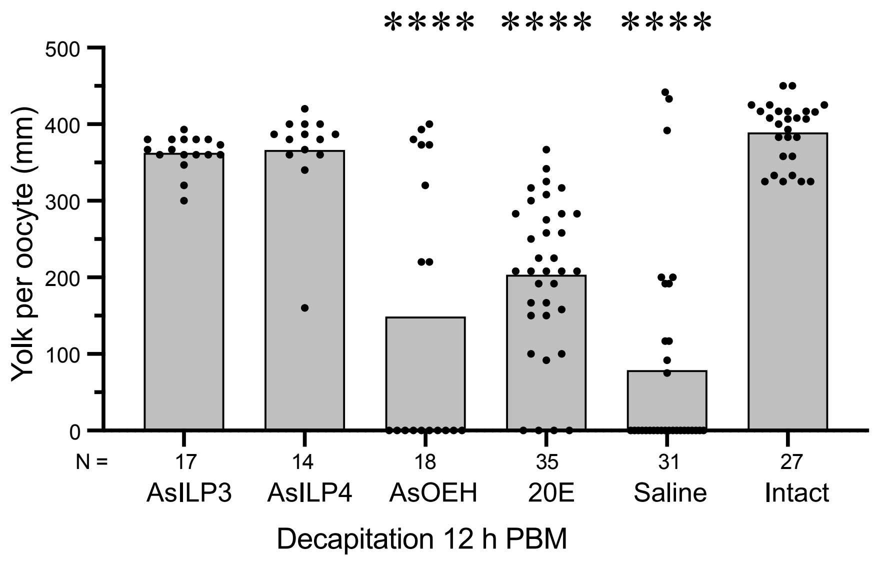

Fig. S4 Yolk deposition into oocytes at 48 h PBM after *An. stephensi* females were blood fed, decapitated 12 h PBM, and injected with AsILP3 (20 pmol), AsILP4 (20 pmol), AsOEH (20 pmol), AaOEH (20 pmol), 20E (1 μmol), or Saline. Yolk deposition into oocytes at 48 h PBM in Intact (non-decapitated) females served as the positive control. Bars in each graph show mean amounts of yolk while solid circles show yolk per independent replicate for each treatment. Total number of replicates (females) analyzed per treatment are indicated below the x axis of each graph. Statistical significance for the treatments shown on each graph was determined after assessing homogeneity of variances followed by a Kruskal-Wallis and a post-hoc Dunn's test. Asterisks above a given bar indicate a given treatment significantly differed from the designated control: $p<0.0001$ (****).

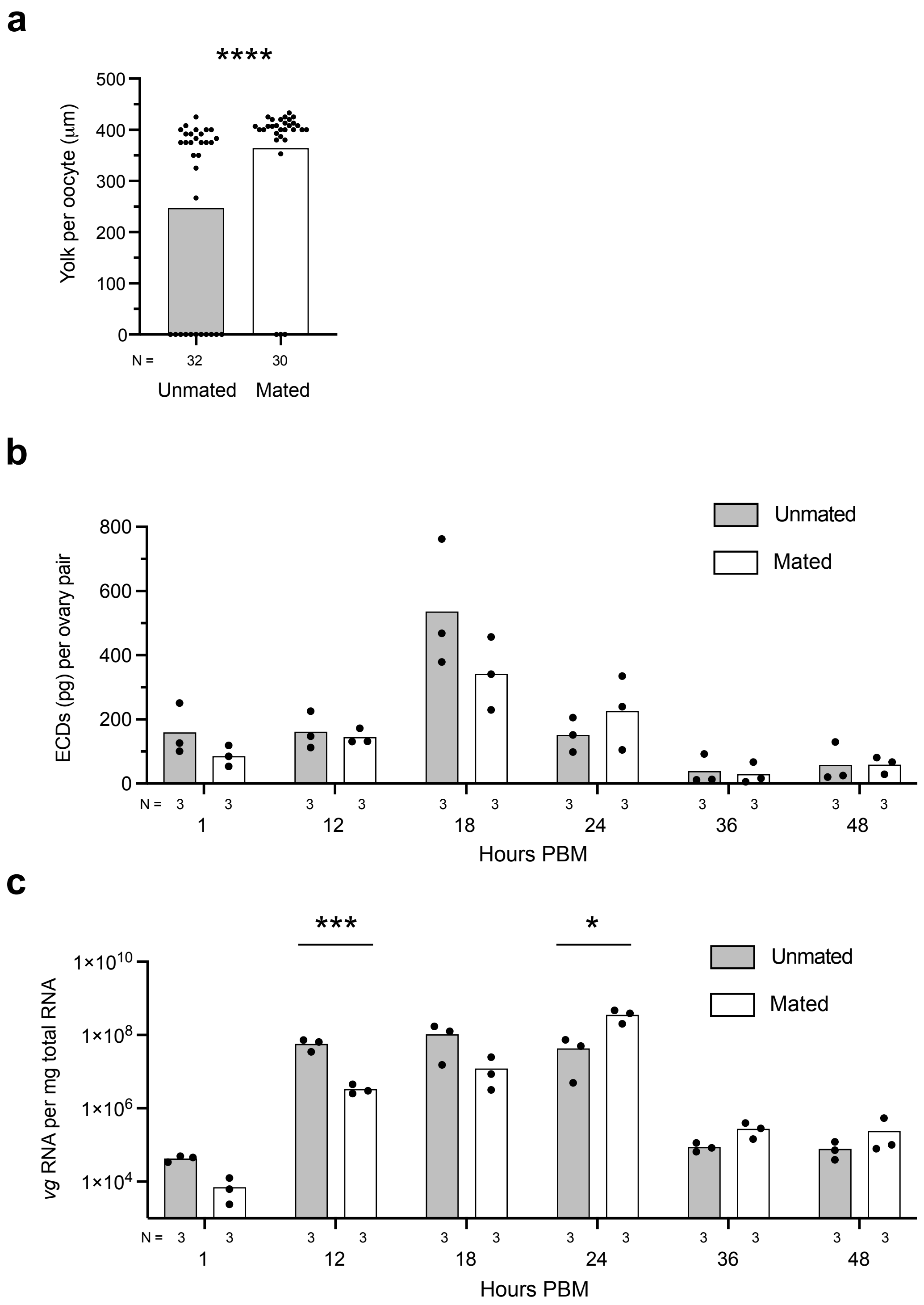

Fig. S5 Mated *An. stephensi* female deposit more yolk into oocytes but show no differences in ECD production by the ovaries or *vg* transcript abundance when compared to unmated females. (**a**) Yolk deposition per oocyte 48 h PBM by unmated and mated females. Bars in each graph

show mean values while solid circles show number of females analyzed per treatment. Total number of females examined per treatment is indicated below the x axis. Statistical significance was determined after assessing homogeneity of variances by a two-tailed Mann-Whitney test. The asterisks above the graph indicate unmated and mated females significantly differed in yolk deposition per oocyte: $p<0.0001$ (****). (**b**) ECD production per ovary pair by unmated and mated females at 1-48 h PBM. (**c**) Transcript abundance for *vg* in unmated and mated females from 1-48 h PBM. Bars in each graph show mean values while solid circles show independent replicates per treatment. Total number of replicates per treatment are indicated below the x axis of each graph. ECD per replicate was determined from two ovary pairs collected from two females: thus, n values on the x axis indicate number of ovary pairs analyzed per treatment. *Vg* per replicate was determined from total RNA that was isolated from a female abdomen in which the ovaries and gut were removed: thus, n values on the x axis indicate number of abdominal pelts analyzed per treatment and time point PBM. Statistical significance for the treatments shown in **b** and **c** was determined after assessing homogeneity of variances followed by an unpaired t-test that compared unmated to mated females at each time point PBM. No differences were detected in ECD production by ovaries from unmated and mated females at any time point. Asterisks in **b** indicate *vg* transcript abundance differed between unmated and mated females at 12 and 24 h PBM: $p<0.05$ (*), $p<0.001$ (***).

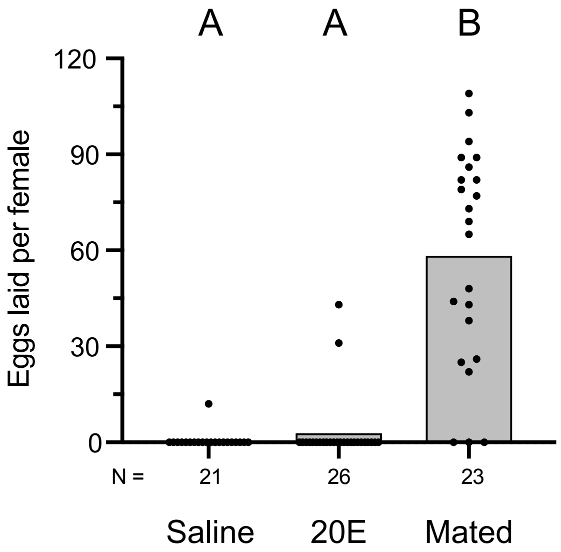

Fig. S6 Number of eggs laid by mated *An. stephensi* females after blood feeding to repletion versus unmated females that blood fed to repletion that were injected with Saline (negative control) or 20E. Note that for these assays, the same mated female data set used in Fig. 8a was used to compare egg laying by unmated females injected with saline or 20E. Total number of females examined per treatment is indicated below the x axis of each graph. Statistical significance was determined after assessing homogeneity of variances followed by a Kruskal-Wallis test and post-hoc Tukey multiple comparison test. Different letters above a given bar indicate number of eggs laid per female significantly differed ($p \leq 0.05$).
